# Essential long-range action of Wingless/Wnt in adult intestinal compartmentalization

**DOI:** 10.1371/journal.pgen.1008111

**Published:** 2019-06-13

**Authors:** Ai Tian, Deepesh Duwadi, Hassina Benchabane, Yashi Ahmed

**Affiliations:** Department of Molecular and Systems Biology and the Norris Cotton Cancer Center, Geisel School of Medicine at Dartmouth College, Hanover, NH, United States of America; University of Michigan, UNITED STATES

## Abstract

Signal transduction activated by Wingless/Wnt ligands directs cell proliferation and fate specification in metazoans, and its overactivation underlies the development of the vast majority of colorectal cancers. In the conventional model, the secretion and movement of Wingless to cells distant from its source of synthesis are essential for long-range signaling in tissue patterning. However, this model was upended recently by an unanticipated finding: replacement of wild-type Drosophila Wingless with a membrane-tethered form produced viable adults with largely normal external morphology, which suggested that Wingless secretion and movement are dispensable for tissue patterning. Herein, we tested this foundational principle in the adult intestine, where Wingless signaling gradients coincide with all major boundaries between compartments. We find that the critical roles of Wingless during adult intestinal development, which include regulation of target gene activation, boundary formation, stem cell proliferation, epithelial cell fate specification, muscle differentiation, gut folding, and signaling crosstalk with the Decapentaplegic pathway, are all disrupted by Wingless tethering. These findings provide new evidence that supports the requirement for the direct, long-range action of Wingless in tissue patterning, with relevance for animal development, tissue homeostasis and Wnt-driven disease.

## Author summary

The evolutionarily conserved Wnt/Wingless signal transduction pathway regulates cell proliferation and cell fate specification during animal development and tissue homeostasis. Deregulation of Wnt signaling triggers the development of nearly all colorectal cancers and is associated with several other diseases. Whereas it is well-established that Wnt ligands are secreted from the cells in which they are produced and move from there to distant cells, whether this movement is essential for Wnt function in cell fate specification has recently become controversial. The goal of this work was to determine whether the movement of Wnt ligands to distant cells is required for tissue patterning. We utilized a genetic model in the Drosophila adult intestine, which like its mammalian counterpart, is subdivided into compartments with distinct anatomy, physiology, functional roles, cell types, and gene expression patterns. Wingless, the primary Drosophila Wnt ligand, is expressed and activates signaling at each major intestinal compartment boundary to specify cell fate. We identified many processes in which Wingless is required for development of the adult intestine, and found that the ability of Wingless to move from its site of synthesis and act at long range is essential. This work demonstrates that the ability of Wingless to signal at long range is indeed critical for tissue formation and function, with relevance for animal development and human disease.

## Introduction

The concept of morphogens—secreted molecules that spread from their source of synthesis and activate signaling in distant cells to direct tissue patterning—was proposed more than a century ago [1,2,3,4,5]. Morphogens directly induce the transcriptional activation of two classes of target genes: high-threshold target genes expressed near the source where morphogen concentrations are at their peak, and low-threshold target genes expressed not only at the source, but also at a distance, where morphogen concentrations are lower. Activation of signaling by morphogens as a function of concentration and distance was proposed to confer precise and robust control of tissue patterning, ensuring proper organ size and shape during development. However, the existence of morphogens and their potential roles in tissue patterning remained controversial for many decades [6,7]; evidence that resolved this controversy was not provided until the mid-1990’s, and was based on landmark studies in Drosophila on the evolutionarily conserved signaling pathways Wingless/Wnt and Decapentaplegic/BMP [8,9,10,11,12,13].

Wnt signaling is essential for growth and patterning of tissues in metazoans and is deregulated in many human diseases [14,15]. The secretion of Wnt ligands from producing cells and their binding to cognate receptors on receiving cells triggers a signal transduction cascade that activates the β-catenin-Tcf transcription complex. Initial studies revealed that Wingless (Wg), a Drosophila Wnt homologue, is essential for long-range patterning of the dorsoventral (DV) boundary region in the adult wing [16,17]; however, the testing of whether Wg functions as a morphogen in this process awaited the development of several experimental tools. First, a Wg antibody revealed both high intensity Wg staining in a narrow stripe of Wg-producing cells at the dorsoventral boundary of the larval third instar wing imaginal disc (the precursor of the adult wing), and lower intensity staining in apparent vesicles that extended at least 10 cell diameters away, supporting the idea that Wg spreads from its source to form a concentration gradient [10,11,13,18]. Second, both high- and low-threshold target genes that are differentially expressed as a function of Wg concentration and distance from its source of synthesis were identified, supporting a role for Wg movement in target gene activation. Critically, target gene activation in cells at a distance from the Wg source was dependent on their continuous ability to receive Wg [13,16]. Third, to inhibit Wg movement, a membrane-tethered form of the Wg protein was generated (NRT-Wg), in which Wg is fused with the transmembrane domain of Neurotactin [13]. Overexpression of wild-type Wg in clones of larval wing disc cells resulted in Wg target gene expression both near and at a distance from this source; in contrast, overexpression of membrane-tethered NRT-Wg also activated expression of the same target genes, but only in cells immediately adjacent to the source. Together, these studies provided compelling evidence that Wg movement establishes a concentration gradient that acts to instruct tissue patterning over a long range, and coupled with concurrent studies on Decapentaplegic, effectively ended the long-standing morphogen controversy [8,9,10,11,12,13].

Two decades after these influential studies, the advent of genome editing enabled revisitation of the requirements for Wg movement through generation of tissues in which wild-type Wg was replaced completely by membrane-tethered NRT-Wg [19]. Due to its integration at the *wg* locus, *NRT-Wg* was expressed via the same enhancer/promoter sequences that normally drive wild-type *wg*. NRT-Wg was detected in the stripe of Wg-producing cells at the dorsoventral wing disc boundary, but not at a distance from this source, in contrast with wild-type Wg [19]. Consistent with previous studies [13], these more recent results provided additional evidence supporting the conclusion that NRT-Wg is membrane tethered. Surprisingly however, tethered Wg was fully capable of producing not only normally patterned wings, but also viable adults with nearly normal external morphology, albeit with a reduced wing size and a significant delay in development [19,20]. These unexpected findings challenged the well-accepted tenet that Wnts must be secreted and move from their source of synthesis to distant cells to direct tissue patterning. Instead, these results suggested that the long-range action of Wg is dispensable for cell fate specification, forcing revision of the textbook model for Wnt pathway activation. Subsequent review articles espoused a new model in which Wnt movement is largely dispensable for tissue patterning, stating that in Drosophila and vertebrate tissues, Wnts act primarily at short range [14,21,22].

**Fig 1:**
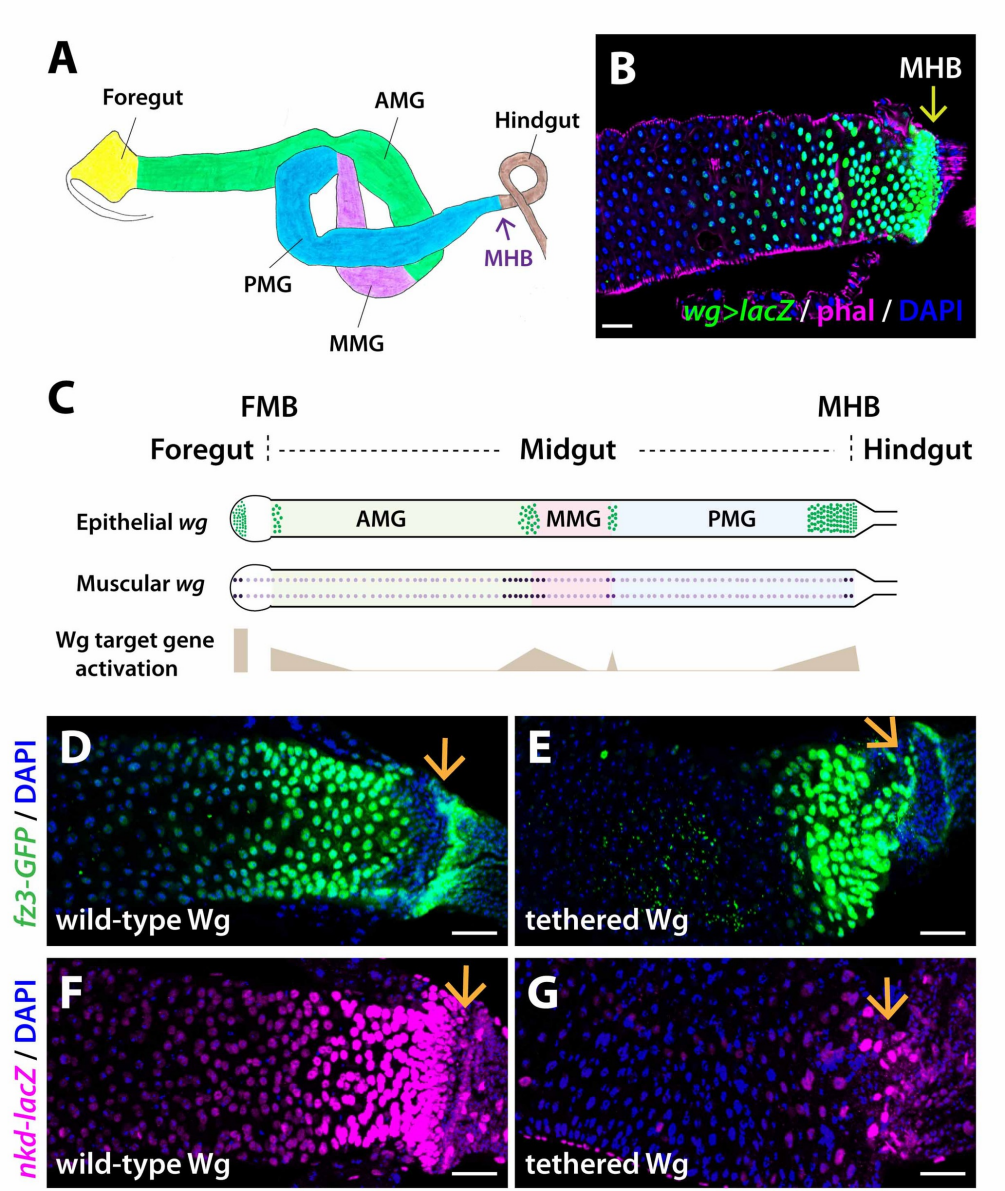
Wg tethering decreases the spatial range of Wg target gene activation. (A) The adult intestine is partitioned into compartments and stereotypical folds. Compartment boundaries separate midgut from foregut and hindgut and further subdivide midgut into anterior midgut (AMG), middle midgut (MMG) and posterior midgut (PMG). MHB: midgut-hindgut boundary. (B) *wg* expression is high at the MHB and decreases as function of distance, revealed by a *wg* transcriptional reporter (*lacZ* expression driven by *Gal4* knocked-in to the *wg* locus). (C) Epithelial and muscle sources of *wg* expression are enriched at major compartment boundaries. Correspondingly, Wg target gene expression peaks at these boundaries and decreases bidirectionally from the source of Wg. (D-G) The spatial range of Wg target gene activation, revealed by *fz3-GFP* and *nkd-lacZ* expression, is diminished when Wg is tethered. wild-type Wg: *wg{KO, Wg-HA}%*; tethered Wg: *wg{KO, NRT-Wg-HA}%*. Anterior, left. Scale bars: (B, D-G) 25 μm.

Given the wide-ranging importance of this fundamental principle in animal development, tissue homeostasis, and disease, we sought to further test the premise that the long-range action of Wg is dispensable for tissue patterning. In particular, as appendages such as the wing might be highly derived, examination of other structures including the internal organs, which rely on different patterning mechanisms, could reveal the general relevance of this principle. We focused on the Drosophila adult intestine, which similar to its mammalian counterpart, is maintained by intestinal stem cells (ISCs) and is subdivided into distinct compartments comprised of different cell types, gene expression profiles, histology, physiology, and function (Fig 1A) [23,24,25,26,27]. Boundaries between intestinal compartments and sub compartments (foregut, midgut, hindgut, and subdivisions thereof) likely act as tissues organizing centers, as lineage analyses revealed that they prevent mixing of cells between different compartments that are defined by discrete domains of gene expression [23,24]. Boundaries also provide the sites for formation of valves and sphincters that control the passage of food. The expression pattern of *wg* in the adult intestine is striking: strong *wg* expression is present at every major compartment boundary, not only in the epithelial cells that line the gut lumen, but also in the overlying visceral muscles that envelop the intestinal epithelium (Fig 1B and 1C) [23,28,29,30,31,32,33]. Consequently, the transcriptional activation of Wg target genes peaks at each of the major compartment boundaries and decreases in a graded manner as a function of distance from these boundaries, extending bidirectionally from the source of Wg-producing cells (Fig 1C) [23,28] to direct cell fate specification during development [28,32,34,35,36]. The transcriptional regulation of Wg target genes at compartment boundaries and within intestinal compartments requires the direct and continuous activation of the Wg pathway, not only near the Wg-producing cells, but also at a distance from this source, as revealed by analysis of null mutant clones of essential Wg pathway components [28,37].

We reasoned that these multiple Wg signaling gradients in the adult intestine, coupled with the genetic tools developed for their analysis, would provide a rigorous model to further test whether the long-range action of Wg is indeed dispensable for tissue patterning. Herein, we identify multiple roles of Wg in patterning and compartmentalization of the adult intestine. We demonstrate that if Wg is tethered, severe defects arise in the spatial range of Wg target gene activation, compartment boundary formation, stem cell proliferation and epithelial fate specification, bundling, striation, and pathfinding of visceral muscle fibers, communication between epithelium and muscle, gut folding, crosstalk with the Dpp signal transduction pathway and organismal viability and fitness. These defects are phenocopied by inhibition of Wg signaling in the developing adult intestinal epithelium. Our findings reveal that inhibition of Wg secretion and movement prevents proper development of the adult gut, and call for reassessment of the requirement for the direct, long-range action of Wg in tissue patterning.

## Results

### Tethering of Wg decreases the spatial range of Wg target gene activation at the midgut-hindgut boundary

*wg* is expressed at the midgut-hindgut boundary (MHB) of the adult intestine (Fig 1B) [23,28]. Similarly, a functional GFP-Wg protein encoded by the endogenous *wg* locus [38] is present at the MHB (S1A and S1A’ Fig). To determine whether Wg tethering alters the spatial range of Wg target gene activation at the MHB, we examined the expression of two target genes that are directly activated by the β-catenin/Tcf transcription complex: *frizzled-3* (*fz3*) [39,40] and *naked* (*nkd)* [41]. We analyzed three transcriptional reporters lines for *fz3* and *nkd*: *fz3-GFP*, in which GFP is inserted at the endogenous *fz3* locus [42], *fz3-RFP*, in which an *RFP* transgene driven by 2.3 kb from the *fz3* enhancer mimics endogenous *fz3* expression [43], and *nkd-lacZ*, in which *lacZ* is inserted at the endogenous *nkd* locus [41]. Similar to *wg*, all three Wg target gene reporters are expressed at the MHB; moreover, their expression decreases as a function of distance from the MHB, forming gradients within the posterior midgut. (Figs 1D and 1F and S1B) [23,28,44]. The *nkd-lacZ* gradient in the posterior midgut extends to at least 60 cell diameters from the MHB [37].

If Wg were to act at long range, then the tethering of Wg would restrict the spatial range of Wg target gene activation. To test this at the MHB, we examined the effects of Wg tethering on the activation of Wg target genes (in homozygous *wg{KO; NRT-Wg}* intestines, herein referred to as *NRT-Wg*). In comparison with controls, the spatial range of Wg target gene activation near the MHB is reduced in *NRT-Wg* intestines, as revealed by the restricted expression of all three transcriptional reporters (Figs 1D–1G and S1B and S1C). We conclude that Wg tethering reduces the spatial range of Wg target gene activation, consistent with the conclusion that Wg normally acts at long range to activate target genes in the posterior midgut adjacent to the MHB.

### Tethering of Wg disrupts visceral muscle patterning at the MHB and posterior midgut

**Fig 2:**
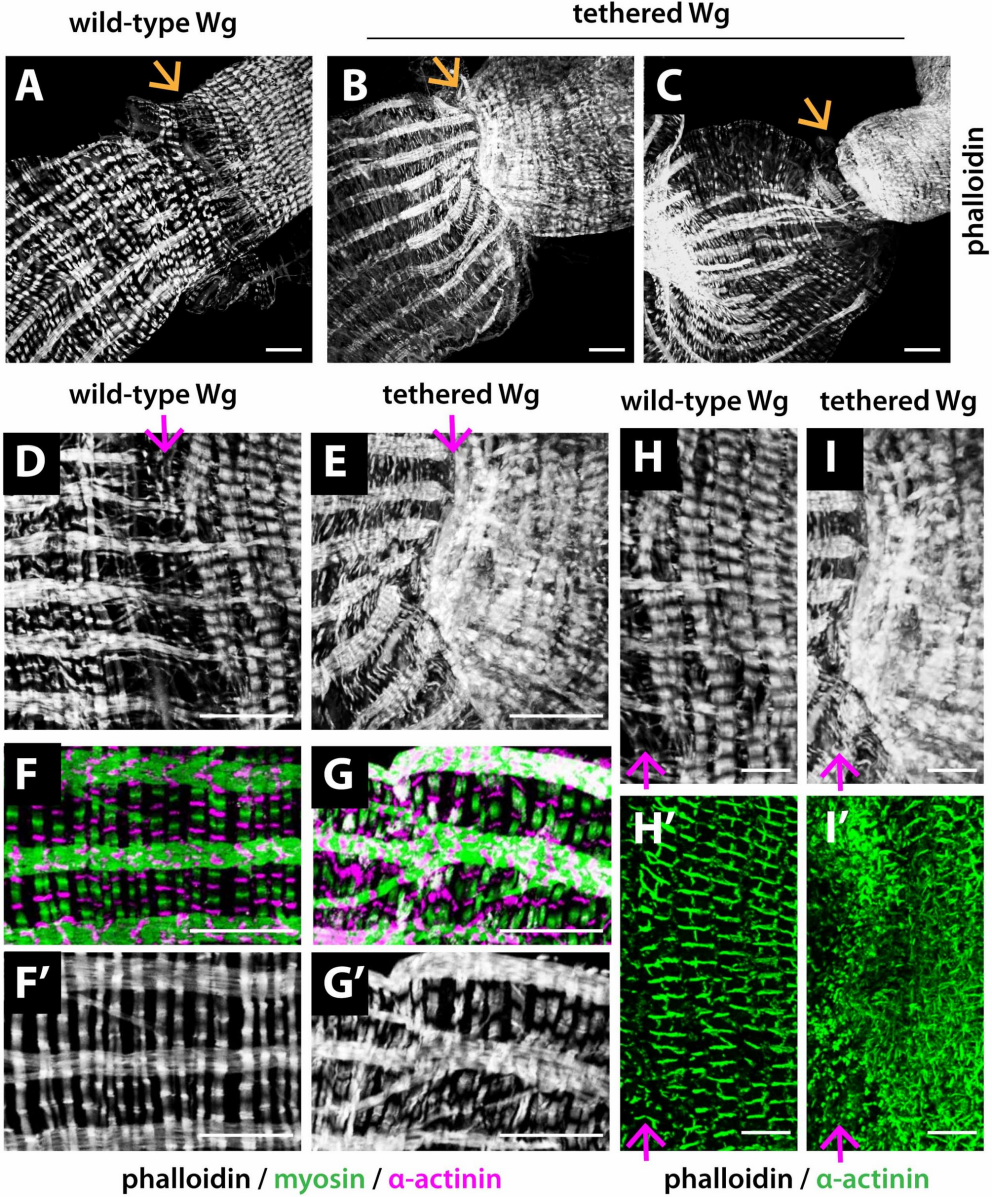
Wg tethering disrupts muscle patterning near the MHB. Phalloidin, which labels myofibrils, reveals the distinct muscle patterns across the MHB. (A) A muscle sheath composed of both longitudinal and circular muscles envelops the midgut epithelium. In contrast, the hindgut epithelium is enveloped primarily by circular muscles. A loosely structured transition zone exists between the midgut and hindgut (arrow). (B-C) The transition zone is lost in *NRT-Wg* intestines, resulting in the direct joining of the midgut and hindgut, with a constriction at the joint (arrow). (D-E) Higher-magnification view of the MHB. The transition zone between midgut and hindgut muscles is lost when Wg is tethered. The longitudinal muscles, instead of being parallel, converge on the now much narrower MHB, while the circular muscles lose their characteristic striated pattern. (F-G’) Extensive breaks and branches in *NRT-Wg* longitudinal muscles within the posterior midgut. In addition, the striated circular muscles, composed of alternating thin fibers (marked by α-actinin and phalloidin) and thick fibers (marked by myosin), are thinner, tightly packed, and wavy with disarrayed banding when Wg is tethered. (H-I’) Striated circular hindgut muscles are thinner and tightly packed with disorganized α-actinin banding in *NRT-Wg* intestines. wild-type Wg: *wg{KO, Wg-HA}%*; tethered Wg: *wg{KO, NRT-Wg-HA}%*. Anterior, left. Arrow marks the MHB. Scale bars: (A-G’) 25 μm, (H-I’) 12.5 μm.

We sought to determine whether the reduced range of target gene activation resulting from Wg tethering alters muscle patterning at the MHB. The wild-type midgut epithelium is enveloped by two types of visceral muscles: longitudinal muscles arranged in parallel arrays and circular muscles that display striated banding with organized thick and thin filaments and distinctive myofibril bundling (Fig 2A and 2D) [45]. In contrast with the midgut, most of the hindgut epithelium is enveloped primarily by circular muscles, which display a pattern of bundling and banding that is distinct from the midgut circular muscles (Fig 2A and 2D) [45]. Moreover, a well-demarcated transition zone exists between the midgut and hindgut, in which thinner muscle fibers surround both the MHB and adjacent rows of midgut and hindgut cells. This transition zone is characterized by a reduction in the number of circular muscles and a reduction in the width of the longitudinal muscles that reach across this region and attach to the anterior most rows of circular muscles in the hindgut.

We observed patterning defects in both the longitudinal and circular muscles at the MHB when Wg was tethered. First, in 80 to 90% of *NRT-Wg* intestines, the transition zone between midgut and hindgut was lost and replaced by direct joining of midgut to hindgut muscles (Fig 2A–2E). In contrast with wild-type, this aberrant junction in *NRT-Wg* intestines was fragile and thus easily torn during dissection. Concomitantly, the diameter of the intestine was reduced at the MHB, and in some *NRT-Wg* intestines, a constriction at this site was accompanied by complete twisting of the intestine along the longitudinal axis (Fig 2A–2E). Second, in *NRT-Wg* intestines, the longitudinal muscles deviated from their normal parallel arrays, bending towards the aberrantly constricted MHB (Fig 2A–2E). In contrast with controls, some longitudinal muscles neither crossed over the MHB, nor made attachments with hindgut circular muscles, nor thinned as they approached the MHB (Fig 2A–2E). In addition, excessive branching or complete breaks were present in longitudinal muscles in the posterior midgut near the MHB (Fig 2A–2G’). Third, the thick and thin filaments in circular muscles in both the *NRT-Wg* midgut and hindgut were disarrayed, as revealed by myosin to mark thick filaments and α-actinin or phalloidin to mark thin filaments (Fig 2F–2I’). In addition, circular muscle fibers in both the *NRT-Wg* midgut and hindgut were improperly bundled and therefore thinner (Fig 2F–2I’). We conclude that Wg tethering prevents proper patterning of visceral muscles at the adult intestinal MHB, and juxtaposed midgut and hindgut regions.

### Tethering of Wg deregulates cell proliferation and fate specification at the MHB and posterior midgut

**Fig 3:**
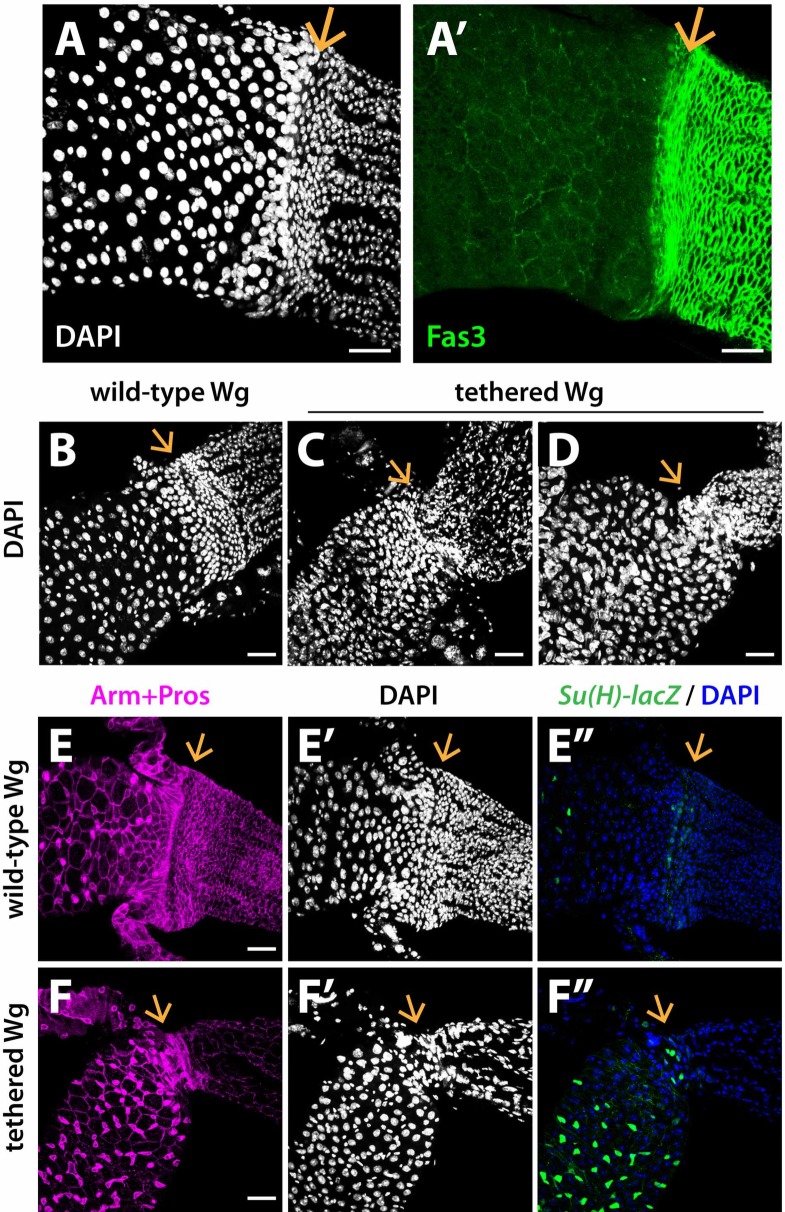
Wg tethering results in severe epithelial defects at the MHB. (A-A’) Midgut and hindgut epithelia exhibit distinct nuclear size, nuclear density and gene expression patterns. Adjoining the MHB, the midgut and hindgut epithelial cells tightly cluster and line against each other, forming an epithelial transition zone. The levels of Fas3, a structural protein, are high in the hindgut but low in the midgut [36]. (B-F”) The epithelial transition zone is lost in *NRT-Wg* intestines. The anterior hindgut contains loosely-packed “ileum-like” cells while supernumerary cells are detected in the midgut. wild-type Wg: *wg{KO, Wg-HA}%*; tethered Wg: *wg{KO, NRT-Wg-HA}%*. Anterior, left. Arrow marks the MHB. Scale bars: (A-F”) 25 μm.

In the epithelium, several distinct features demarcate the MHB, where the endoderm-derived midgut is juxtaposed with the ectoderm-derived hindgut [29,32,36,46]. The marked differences that exist in the midgut, hindgut, and transition zone epithelia with respect to cell fate, nuclear and cell size, and gene expression profiles (Fig 3A and 3A’) reflect their distinct origins. The midgut epithelium is distinguished by large enterocytes with polyploid nuclei that function in absorption of nutrients. In contrast, small, quiescent epithelial cells with diploid nuclei comprise the hindgut epithelium, and function primarily in water absorption (Fig 3A). The unique epithelial structure at the MHB arises from closely-spaced terminal posterior midgut cells that abut the pylorus, which is the anterior most compartment in the hindgut (Figs 1A and 3A). Another distinguishing feature is the distinct gene expression profiles in the midgut versus hindgut epithelium, as represented by Fas III, levels of which are low in the midgut, increase sharply at the MHB, and are high in the hindgut (Fig 3A’) [36].

When Wg is tethered, several defects in epithelial structure were evident at the MHB, midgut, and hindgut. For example, the epithelial transitional zone at the MHB was absent in 80–90% *NRT-Wg* intestines, and replaced by direct linkage of midgut to hindgut (Fig 3B–3F’). In addition, the hindgut epithelium was disorganized and shrunken (Fig 3B–3D, 3E’ and 3F’). Moreover, anterior to the MHB an increase in the diameter of the posterior midgut and an increased number of epithelial cells were observed (Fig 3B–3D, 3E’ and 3F’). To identify these supernumerary cells, we labelled progenitor cells with *Stat-GFP* [47] and enteroblasts with *GBE-Su(H)-lacZ* [25]. We found that the increased number of cells near the MHB included both progenitors and enterocytes (Figs 3E’, 3E”, 3F’ and 3F” and 1D and 1E), suggesting that ISCs overproliferated in *NRT-Wg* midguts. These findings reveal that Wg tethering deregulates cell proliferation and fate specification during formation of the epithelium of the adult intestinal MHB and surrounding midgut and hindgut.

We next addressed whether the possibility of ‘cellular memory’ of signaling that originates during a prior developmental stage (see Discussion) contributes to *NRT-Wg* adult intestinal phenotypes. First, we examined the *NRT-Wg* larval gut and observed no defects in either the structure of the epithelium at the MHB or in progenitor cell clusters in the adjacent posterior midgut, as revealed by Fas III and Armadillo staining (S2A–S2D” Fig). These findings suggest that epithelial defects in *NRT-Wg* arise during formation of the adult intestine during pupation, as they were not present in the *NRT-Wg* larval intestine. To test this conclusion, we used a conditional allele [19] to convert the endogenous *wg* locus from expressing wild-type Wg-HA to expressing NRT-Wg-HA during development of the adult intestine, which occurs in pupation. We first examined both wild-type Wg-HA and NRT-Wg-HA, which were detectable at the MHB with antibodies directed against the HA epitope tag (S3A and S3B’ Fig); NRT-Wg-HA staining was more intense than the staining of wild-type *Wg-HA*, as previously documented in the larval wing disc [19]. Next, we found that conversion from the wild type Wg-HA allele to the *NRT-Wg-HA* allele during pupation phenocopied the intestinal epithelial phenotype observed in homozygous *NRT-Wg* adults: disruption of patterning was observed at the MHB and posterior midgut (S3D Fig). Together, these results indicate that putative cellular memory of Wg signaling in the larval intestine is not sufficient to direct development of the adult gut, consistent with the observation that activation of Wg target genes at compartment boundaries and within intestinal compartments requires continuous Wg pathway activation [28,37].

### Tethering of Wg disrupts gut folding near the MHB

**Fig 4:**
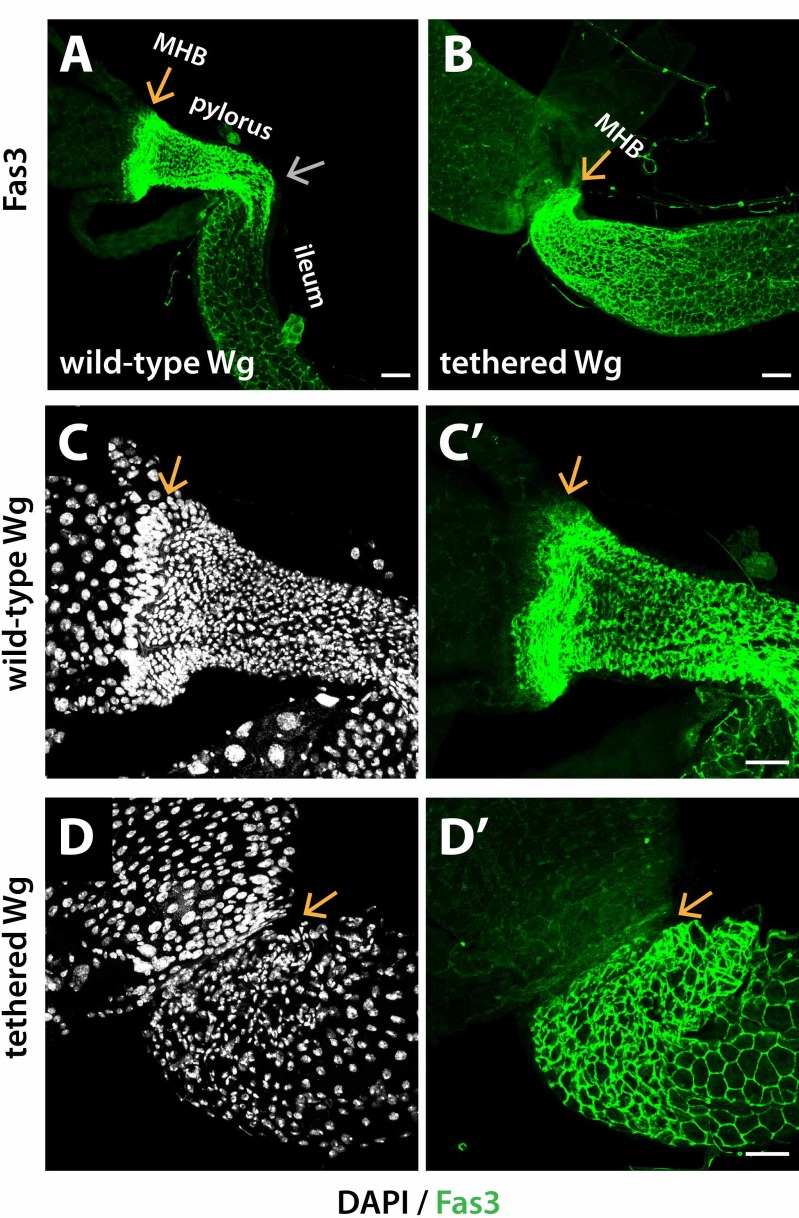
Wg tethering disrupts gut folding near the MHB. (A-B) The characteristic fold that separates the pylorus and ileum, as well as the primary region of the pylorus is lost when Wg is tethered. (C-D’) The majority of the tightly-packed pylorus cells are lost in *NRT-Wg* mutants. wild-type Wg: *wg{KO, Wg-HA}%*; tethered Wg: *wg{KO, NRT-Wg-HA}%*. Orange arrow marks the MHB. Silver arrow marks the fold between the pylorus and ileum. Anterior, left. Scale bars: (A-B) 50 μm, (C-D’) 25 μm.

Within the abdominal cavity, the adult intestine displays a characteristic pattern of folds and loops along its length [23] (Fig 1A). Posterior to the MHB, the diameter of the hindgut decreases gradually, followed by formation of a signature fold in the hindgut (Fig 1A). This fold marks the transition between two hindgut compartments: the posterior pylorus, which contains a sphincter and valve to control the passage of food and displays high levels of Fas III and Arm, and the anterior ileum [29,32], which is distinguished from the pylorus by its larger diameter, larger epithelial cells, and lower levels of Fas III and Arm (Figs 4A and S1F and S1F’). Thus, this unique region includes a major inflection point of the gut in the abdominal cavity and also a boundary separating two distinct hindgut compartments.

In controls, this hindgut fold was retained following dissection and fixation of the intestine (Figs 4A and S1F and S1F’). However, in *NRT-Wg*, the fold was lost, as was the majority of the pylorus, and replaced by an apparent direct joining between the midgut and ileum-like tissue (Figs 4A and S1F and S1G’). Therefore, we conclude that Wg tethering prevents formation of folds near the MHB and also promotes ectopic twists at this site (Fig 2C).

### Signaling activity of tethered Wg at the source of synthesis

Is signaling activity reduced in NRT-Wg? If so, is this reduced activity sufficient to pattern the wing disc but not the adult intestine? We took two approaches to test the possibility that *NRT-Wg* phenotypes in the adult intestine result from decreased NRT-Wg signaling activity at the source of synthesis, rather than from the block of Wg secretion. First, we hypothesized that if *NRT-Wg* gut phenotypes result from reduced signaling activity, then these defects might be rescued by overexpression of *NRT-Wg*. To test this hypothesis, we used the UAS/Gal4 system coupled with a gene conversion technique [19,48] to overexpress *UAS-NRT-Wg* in animals that simultaneously expressed a ‘flipped in’ *NRT-Wg-HA* allele at the endogenous *wg* locus. Previous work revealed that *UAS-NRT-Wg* is active [13]. Immunostaining and quantitation revealed that, by comparison to levels of the flipped in NRT-Wg, overexpressed NRT-Wg levels were at least four-fold higher; this difference in levels was beyond the linear range detectable by confocal microscopy (S3C–S3E’ Fig). Despite the high levels of NRT-Wg protein, no rescue of the intestinal phenotypes was observed (S3D and S3E Fig).

Second, we hypothesized that if signaling activity is reduced in NRT-Wg, then the level of Wg target gene activation would also be reduced, not only at a distance from the source due to tethering, but also near the Wg-producing cells at the intestinal compartment boundaries. To test this hypothesis, we quantitated expression levels of a Wg target gene reporter (*fz3-GFP*) in animals expressing only wild-type Wg-HA versus those expressing only NRT-Wg-HA at two different boundaries: the midgut-hindgut boundary (MHB) and the middle midgut-posterior midgut (MMG-PMG) boundary (S3F Fig). We found that the levels of Wg target gene activation at the boundaries were the same (at the MHB) or greater (at the MMG-PMG boundary) in NRT-Wg intestines as compared with controls. Thus, Wg target gene activation was not reduced at the source in *NRT-Wg* intestines. These results provide evidence that despite the addition of the Neurotactin transmembrane domain, NRT-Wg is capable of high-level signaling activity. These results are consistent with previous analysis of *fz3-GFP* activation at the dorsoventral boundary of *NRT-Wg* wing imaginal discs, which revealed both normal levels of target gene expression near the Wg source and a decreased spatial range of activation in cells distant from the source [19]. Together, these findings support the conclusion that the short signaling range observed in NRT-Wg guts is not due to reduced signaling activity at the source of synthesis.

### Inhibition of Wg signaling phenocopies Wg tethering at the MHB

**Fig 5:**
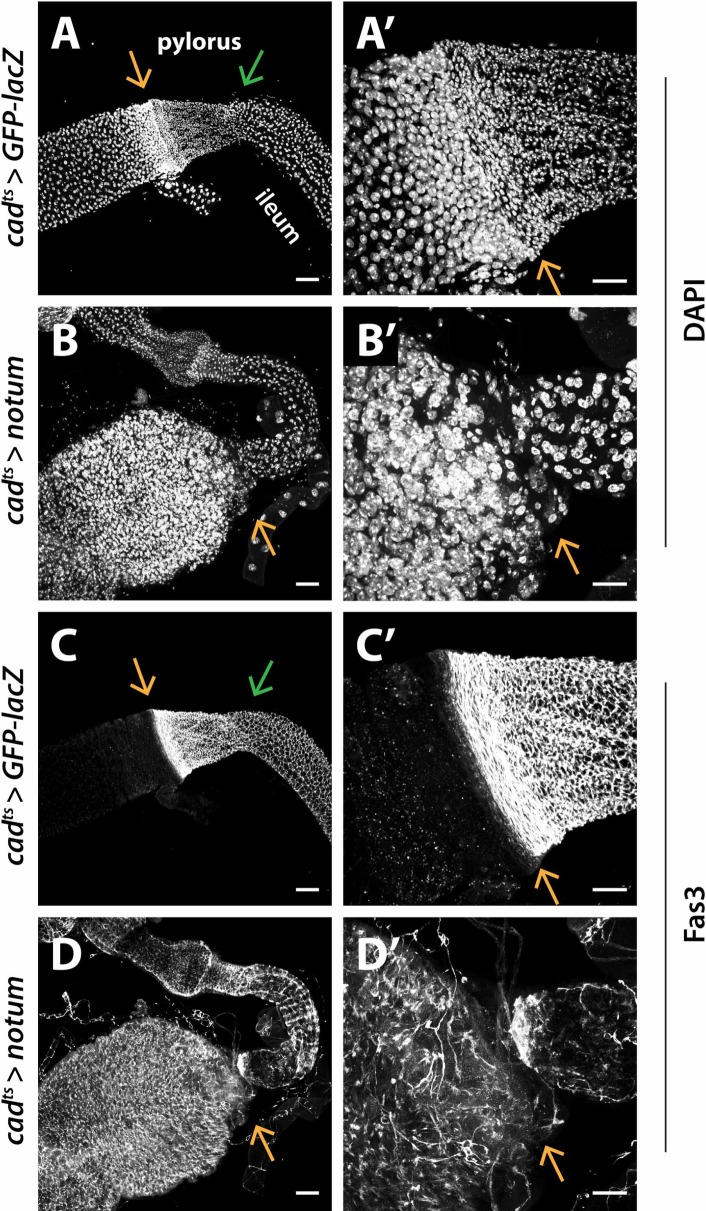
Inhibition of Wingless activity phenocopies the epithelial defects resulting from the tethering of Wg. (A-B’) The transition zone at the MHB is lost upon inhibition of Wg by overexpression of *notum*. Tightly clustered cells with condensed nuclei are detected in both midgut and hindgut. (C-D’) Fas III expression is no longer restricted to the hindgut, replaced by expression both in the midgut and the hindgut. In addition, the fold between the pylorus and ileum is no longer evident. Orange arrow marks the MHB. Green arrow marks the fold between pylorus and ileum. Anterior, left. Scale bars: (A, B, C, D) 50 μm, (A’, B’, C’, D’) 25 μm.

Studies in the larval wing disc suggested that Wg tethering has two effects: Wg accumulates in producing cells and is not detectable at cells away from the source of synthesis [19]. Therefore, we reasoned that Wg gain-of-function at the source, Wg loss-of-function at a distance from the source, or a combination of both might result in the muscle and epithelial cell defects observed in *NRT-Wg* intestines. To distinguish between these possibilities, we either inhibited or increased Wg signaling near the MHB during adult gut development. We used *cad-Gal4*, which drives expression in the epithelium at the MHB and adjacent posterior midgut (S4A Fig), to overexpress a negative regulator of the pathway, Notum. Notum is an extracellular deacylase that inhibits signaling by removing a lipid modification from Wg that is essential for its secretion and interaction with the Frizzled receptor [49,50]. We observed multiple defects in the epithelium following overexpression of *notum*, which qualitatively resembled the defects observed in *NRT-Wg* intestines, but were more severe. First, the epithelial transition zone at the MHB was lost (Fig 5A and 5B’), as was the fold between pylorus and ileum (Fig 5C and 5D’). The entire pylorus was also lost, replaced by what appeared to be a direct joining of the midgut to the ileum. Furthermore, the midgut width was increased, as were supernumerary cells. Phospho-histone H3 (pH3) staining revealed an increase in the mitotic index, indicating excess ISC proliferation in the midgut (S4B Fig). In addition, the levels of Fas III, which are normally low in the midgut, were increased, indicating deregulation of compartment-specific gene expression (Fig 5C and 5D’). Similar defects were observed upon expression of dominant negative TCF with *cad-Gal4*, but were less severe (S4B Fig) [51]. In contrast, overexpression of *wg* using the same *cad-Gal4* driver did not disrupt patterning at the MHB (S4C–S4F” Fig). Thus, inhibition of Wg signaling results in multiple defects in gut development that phenocopy Wg tethering at the MHB epithelium, suggesting that the long-range action of Wg is essential for its role in epithelial patterning at the MHB and the adjacent midgut and hindgut regions.

**Fig 6:**
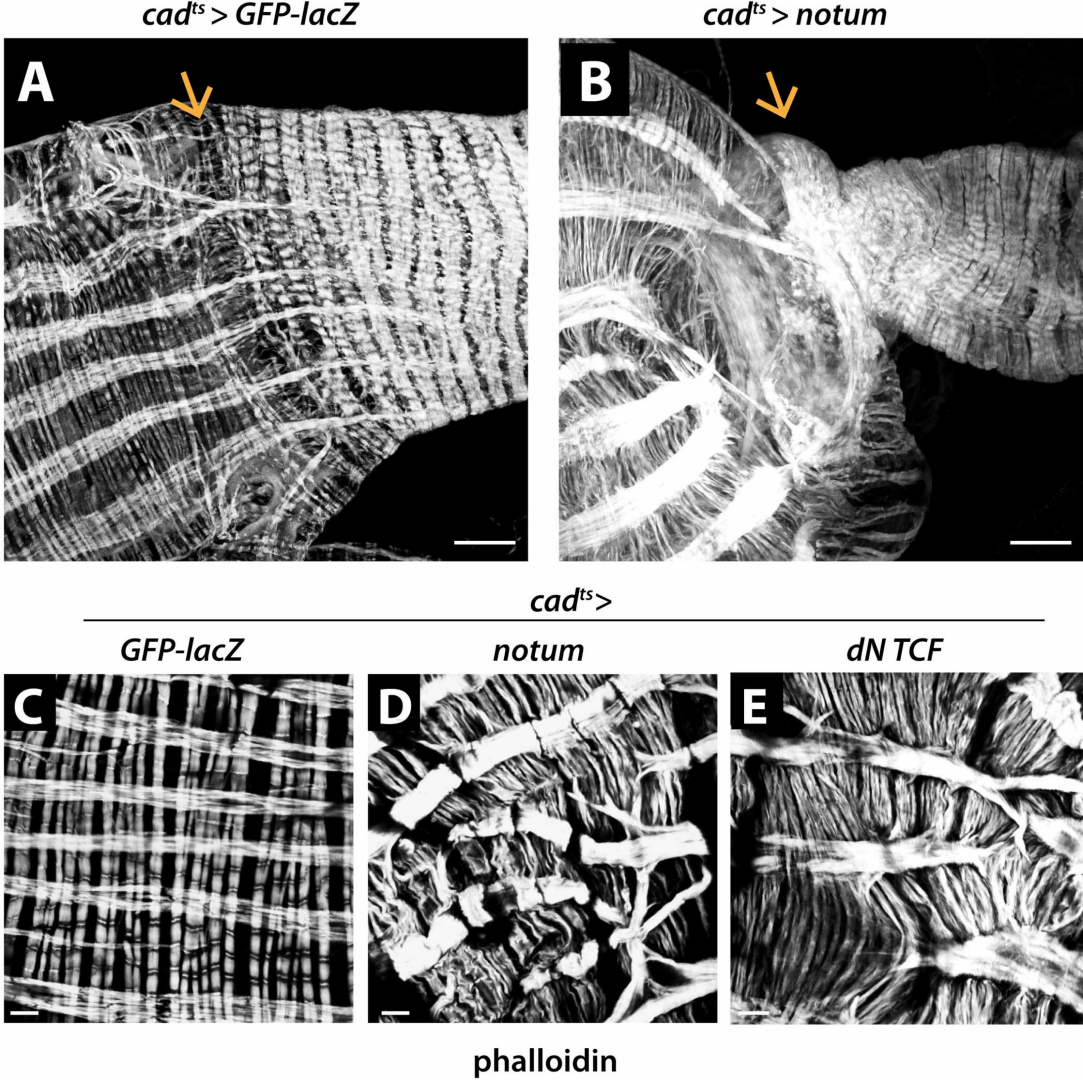
Inhibition of Wg signaling phenocopies muscle defects caused by Wg tethering. (A-B) The muscle transition zone at the MHB is lost upon inhibition of Wg activity resulting from overexpression of *notum*. (C-E) Extensive breaks and branches are detected in longitudinal muscles. The striated pattern of circular muscles is lost, and the muscles are wavy and tightly packed. Overexpression of dominant-negative TCF driven by the epithelial *cad-Gal4* driver gives rise to similar muscle defects. Arrow marks the MHB. Anterior, left. Scale bars: (A-B) 25 μm, (C-E) 10 μm.

Notably, upon overexpression of *notum*, patterning defects were present not only in the epithelium, but also in the overlying visceral muscle. The muscle transition zone at the MHB was lost, and replaced by direct joining of midgut and hindgut muscles (Fig 6A and 6B). Extensive breaks and branches were observed in the longitudinal muscles, and the striated banding pattern of circular muscles was lost (Fig 6A–6D). These defects qualitatively phenocopied the effects of Wg tethering, but were more severe. Furthermore, as *cad-Gal4* induces *notum* expression in the epithelium, the resulting muscle phenotypes suggested that Wg signaling in the gut epithelium is important for patterning the overlying muscle. We sought to test this conclusion using an independent approach, as Notum is a secreted factor [52,53], and thus has the potential to diffuse from the epithelium to the muscle. Therefore, we examined intestines expressing dominant negative *TCF* using the *cad-Gal4* driver during adult gut development (Fig 6E). Notably, similar muscle defects were observed. In contrast, overexpression of Wg did not disrupt muscle patterning at the MHB (S4F and S4F” Fig). These findings support two conclusions. First, the defects arising from Wg tethering at the MHB result from reduction in Wg signaling. Second, patterning of the overlying visceral muscle is dependent on Wg signaling in the MHB epithelium.

### Tethering of Wg prevents Wg-Dpp signaling crosstalk at the MHB

**Fig 7:**
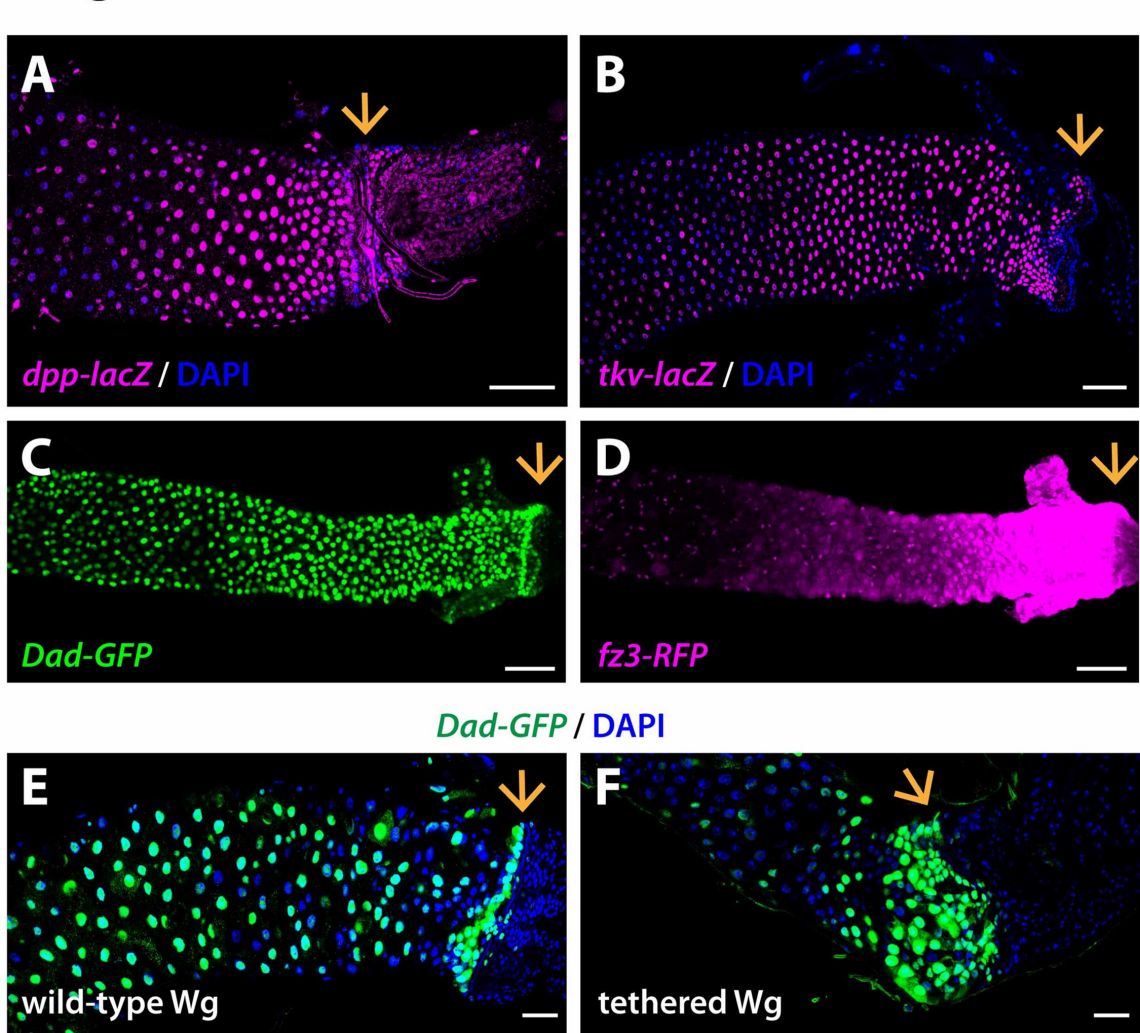
Wg tethering prevents crosstalk between the Wg and Dpp pathways. (A) *dpp-lacZ*, a transcriptional reporter for *dpp*, is expressed in a graded pattern near the MHB, with highest levels at the MHB. (B) *tkv-lacZ*, a transcriptional reporter for the Dpp receptor *Tkv*, is also expressed in a graded pattern near the MHB, with highest levels at the MHB. (C-D) *Dad-GFP*, a transcriptional reporter for the Dpp target gene *Dad*, is expressed in graded pattern near the MHB, similar to the Wg target gene reporter *fz3-RFP*. (E-F) Wg tethering decreases the spatial range of expression of *Dad-GFP*. wild-type Wg: *wg{KO, Wg-HA}%*; tethered Wg: *wg{KO, NRT-Wg-HA}%*. Arrow marks the MHB. Anterior, left. Scale bars: (A-D) 50 μm, (E-F) 25 μm.

Extensive crosstalk exists between the Wg and Dpp pathways in developmental contexts and disease conditions [54,55], and potentially acts to increase precision and robustness in tissue patterning under the temporal constraints of development. Therefore, we sought to determine whether Wg tethering prevents signal pathway crosstalk at the MHB. Both *dpp* and the Dpp target gene *Daughters against dpp* (*Dad)* are expressed at the MHB [56,57](Fig 7A and 7C). Similarly, *thick veins (tkv)*, which encodes a type I Dpp receptor, is also expressed at the MHB (Fig 7B). *dpp* expression peaked at the MHB and decreased in a graded manner as a function of distance from the MHB (Fig 7A), as did the expression of *tkv* and *Dad* (Fig 7B and 7C). As the graded expression patterns of *dpp*, *tkv* and *Dad* were similar to that of Wg target genes (Fig 7D), we hypothesized that Wg signaling induces their expression. To test this hypothesis, we inactivated the essential Wg pathway components, *dishevelled (dsh)* and *pygopus (pygo)*, in mitotic clones of cells near the MHB. We found that *dpp* expression was eliminated in *pygo* null mutant clones at a distance from the MHB (S5A–S5C’ Fig) [58]. Similarly, both *tkv* and *Dad* expression were decreased in *dsh* null mutant clones (S6 Fig) [59]. Conversely, *dpp* expression was aberrantly increased in clones of cells in which Wg signaling was hyperactivated by concomitant inactivation of the negative regulatory paralogs *Apc1* and *Apc2* (S5D–S5E” Fig) [60,61,62,63]. We conclude that Wg pathway activation near the MHB and the adjacent posterior midgut is essential for the expression of *dpp*, *tkv* and Dpp target genes in this region.

Building on this conclusion, we tested whether Wg tethering would alter activation of the Dpp pathway at the MHB. We found that the spatial range of expression of the Dpp target gene *Dad* near the MHB was reduced in *NRT-Wg* intestines (Fig 7E and 7F). We conclude that Wg tethering prevents Dpp target gene activation near the MHB, and therefore prevents crosstalk between the Wg and Dpp signaling pathways in this region.

### Tethering of Wg disrupts development of the MMG

**Fig 8:**
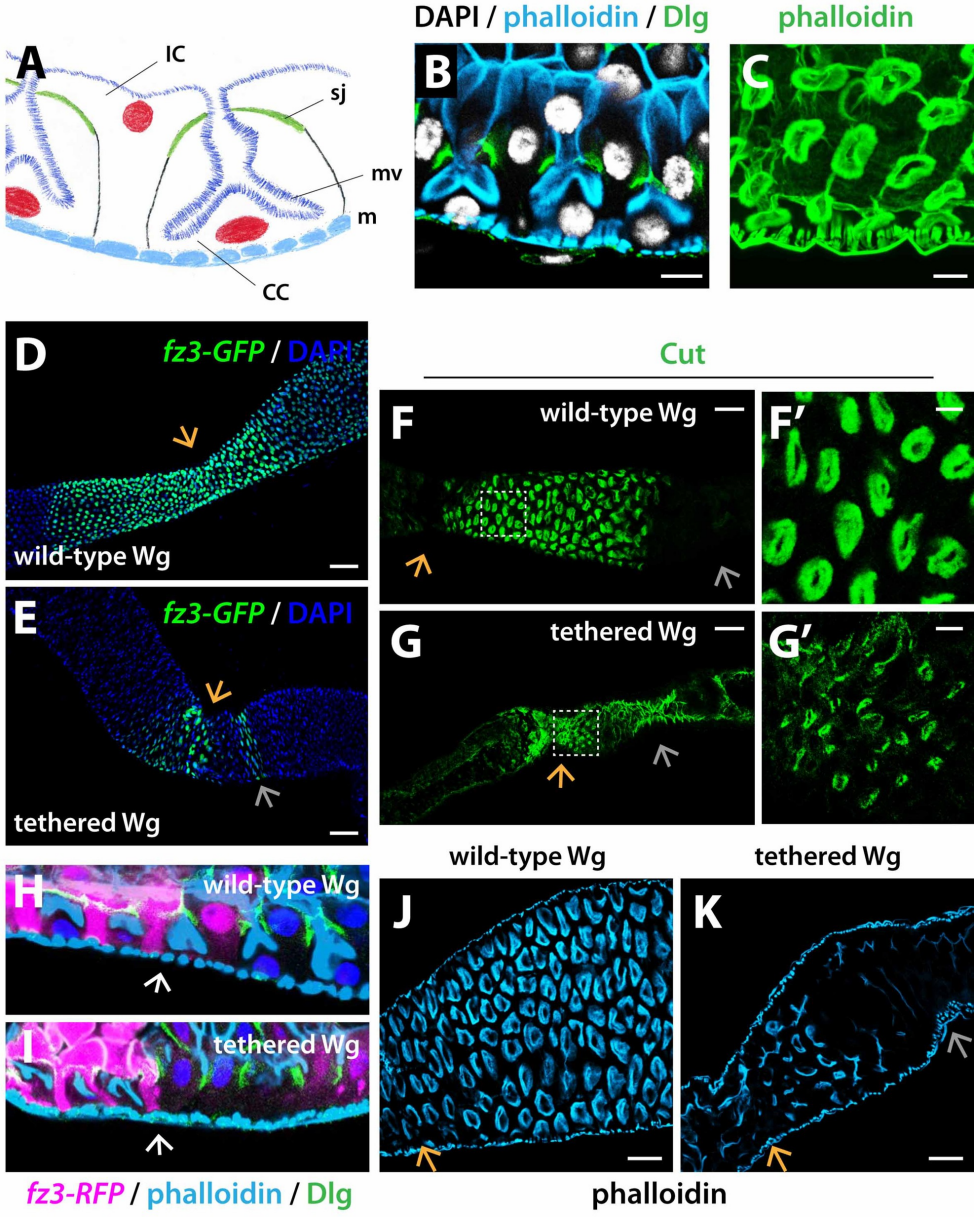
Wg tethering disrupts patterning of the MMG epithelium. (A-C) Acid-secreting copper cells of the MMG. The apical membrane of the copper cells is deeply invaginated, with proton pump-containing microvilli. CC: copper cell; IC: interstitial cell; sj: septate junction; mv: microvilli; m: visceral muscle. Dlg marks the septate junctions between CC and IC. Phalloidin marks the cup-shaped surface formed by CC microvilli, and also the cell cortex and muscle. B, tangential section. C, horizontal section at the level of the cup formed by microvilli (D-E) The spatial range of Wg target gene activation at the anterior boundary of the MMG, revealed by *fz3-GFP* expression, is diminished when Wg is tethered. (F-G’) Wg tethering results in decreased MMG size and decreased number of copper cells. The remaining copper cells have a reduced microvillar surface, revealed by Cut. (H-I) Tangential section of the MMG: few copper cells are present in *NRT-Wg* intestines, and their microvilli structure is poorly differentiated, revealed by phalloidin. (J-K) Top view of the MMG, few copper cells remain and have abnormal shape and structure in *NRT-Wg* intestines, revealed by phalloidin. wild-type Wg: *wg{KO, Wg-HA}%*; tethered Wg: *wg{KO, NRT-Wg-HA}%*. Anterior, left. Orange arrow marks the anterior boundary of the MMG. Silver arrow marks the posterior boundary of the MMG. Scale bars: (B, C, F, G) 10 μm, (D-E) 50 μm, (J-K) 25 μm.

Is the requirement for the long-range action of Wg in the adult intestine limited to the MHB? To address this question, we also analyzed the middle midgut region (MMG), a compartment flanked at both its boundaries by Wg signaling gradients (Fig 1C) [23,28]. The MMG contains acid-secreting copper cells, which are distinguished by their extensive invagination of apical microvilli containing proton pumps (Fig 8A–8C) [64,65,66]. We identified copper cell microvilli using phalloidin or Cut staining, and their septate junctions with juxtaposed interstitial cells using Discs large staining. In *NRT-Wg* intestines, the number of copper cells in the MMG was reduced, with the few remaining copper cells restricted to the anterior midgut/middle midgut boundary (AMB) (Figs 8D–8K and S7). Furthermore, Cut and phalloidin staining were reduced, revealing defects in both copper cell number and differentiation. Together, these findings indicate that Wg tethering prevents patterning of the MMG, in a manner that is qualitatively similar to its effects at the MHB.

**Fig 9:**
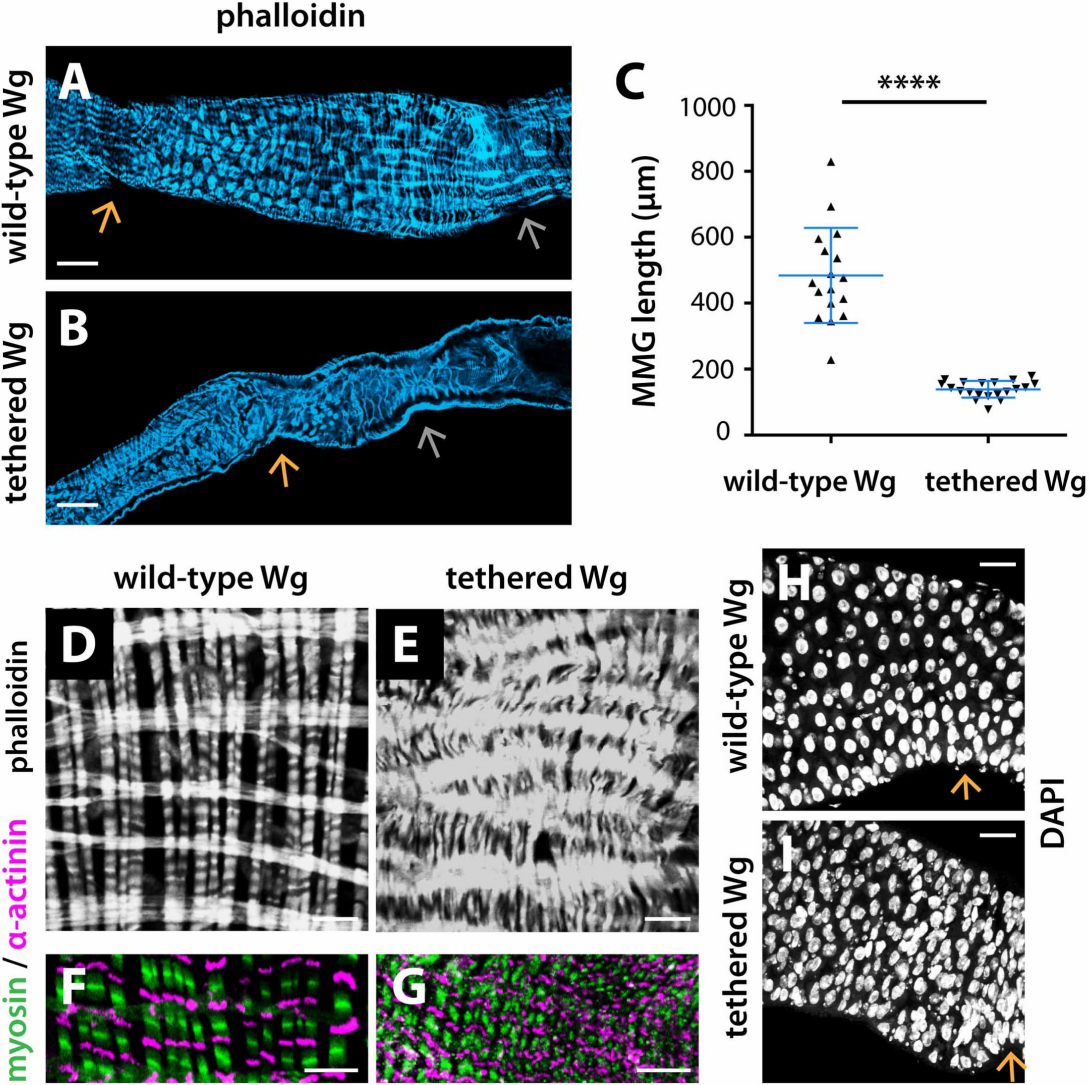
Wg tethering disrupts MMG size and muscle patterning. (A-C) Wg tethering results in decreased MMG size compared to controls. **** P<0.001 (t-test). (D-G) The visceral muscles are malformed in the *NRT-Wg* MMG. (H-I) Aberrant accumulation of cells anterior to the anterior boundary of the *NRT-Wg* MMG. wild-type Wg: *wg{KO, Wg-HA}%*; tethered Wg: *wg{KO, NRT-Wg-HA}%*. Anterior, left. Orange arrow marks the anterior boundary of the MMG. Silver arrow marks the posterior boundary of the MMG. Scale bars: (A-B) 50 μm, (D-G) 10 μm, (H-I) 25 μm.

In addition, the entire MMG was malformed in *NRT-Wg* intestines. First, the MMG was significantly smaller than in controls (Fig 9A and 9B, quantification in Fig 9C), suggesting that Wg tethering prevents MMG growth. Second, the characteristic striation and banding pattern of the circular muscles was lost, with defects in myofibrils bundling (Fig 9D–9G). Moreover, supernumerary epithelial cells accumulated anterior to the AMB (Fig 9H–9I). These findings recapitulated the visceral muscle and epithelial defects observed at the *NRT-Wg* posterior midgut (Figs 2 and 3).

Both inhibition and activation of Wg signaling at the MMG phenocopied *NRT-Wg*. We used *cad-Gal4*, which drives expression at the MMG boundaries at weak levels (S8A Fig), and *dve-Gal4*, which drives strong expression across the whole MMG and its two boundaries (S6B Fig) [66]. Overexpressing *notum* using a temperature-sensitive *cad-Gal4* driver resulted in a reduced size of the MMG (S8C and S8D Fig). The few copper cells that were present exhibited rudimentary microvilli, and the overlying muscles were disorganized (Fig 8E and 8F). The striated banding and bundling of the myofibrils were lost in the circular muscles. Qualitatively similar, but more severe defects were observed upon *notum* overexpression with the *dve-Gal4* driver (S8G and S8H Fig). In contrast with wild-type, *fz3-RFP* expression was restricted to only one region, and reduced in intensity. Structural aberrations in the intestine prevented precise definition of the MMG. Few copper cells were observed, and these displayed abnormal morphologies. These findings suggested that Wg signaling is essential in the MMG.

To further test this conclusion, we performed three additional knockdown experiments. RNA interference (RNAi)-mediated depletion of *wg* using the temperature sensitive *wg-Gal4* driver resulted in reduced MMG size, a phenotype that was recapitulated by knockdown of *wntless (wls)*, which encodes a transmembrane protein required for Wg transport and secretion [67,68,69] (S9A–S9E Fig). Supporting these knockdown data, MMG size was also reduced in *wls* hypomorphic mutants (S9F–S9H Fig) [68]. Together, these observations support the conclusion that reduction in Wg activity phenocopies the tethering of Wg at the MMG, though overexpression of *wg* with *cad-gal4* also resulted in similar patterning defects (S9I and S9J Fig). These studies reveal that a precise level of Wg signaling is essential for MMG development, and is disrupted by Wg tethering.

### Tethering of Wg reduces viability and fitness

*NRT-Wg* homozygotes did not survive when raised at the standard temperature of 25°C, even in uncrowded conditions, revealing increased lethality under growth conditions that are normally optimal. To rule out background effects from other mutations on the *NRT-Wg* chromosome, we repeated these experiments with *NRT-Wg/wg*^*null*^ transheterozygotes, which displayed similar viability defects, suggesting that this defect results from the tethering of Wg. However, some individuals of both genotypes survived to adulthood when the temperature was lowered to 21–22°C to allow for increased developmental time; thus, the *NRT-Wg* flies we analyzed throughout this study were likely the escapers with the least severe developmental defects. Nonetheless, on a standard diet, a reduction in lifespan was observed in the surviving *NRT-Wg* adults as compared with controls (S10A Fig). This reduced lifespan was exacerbated with a sucrose-only diet, on which most *NRT-Wg* adults died within one week (S10B Fig). In addition, the crop was enlarged in *NRT-Wg* intestines (S10C and S10D Fig), suggesting a functional impairment in peristalsis. Consistent with these observations, targeted inhibition of Wg signaling in the gut epithelium similarly reduced lifespan (S10E Fig). The majority of adults overexpressing *notum* or dominant-negative *TCF* in the intestinal epithelium died by one day after eclosion, indicating that Wg signaling in the gut epithelium is indispensable for survival (S10E Fig). Together, these results reveal that Wg tethering reduces viability and fitness.

## Discussion

The development of a tethered Wg protein that retains the ability to activate expression of low and high-threshold Wg target genes [13], and its subsequent encoding at the endogenous *wg* locus [19], were technical breakthroughs in the quest to elucidate the requirement for the long-range action of Wg. However, previous analyses of tethered Wg in the larval wing disc were interpreted to support two diametrically opposed models, leading to controversy regarding whether the long-range action of Wg is necessary for tissue patterning. Given the importance of this fundamental principle, we opted to test the effects of Wg tethering in a distinct physiological context: the adult intestine. In contrast with the larval wing disc, the adult gut forms through distinct developmental mechanisms that arise during metamorphosis [23,70], and faces different functional demands throughout life, including the ability of its epithelium to continuously turnover during homeostasis and to regenerate following injury [23,71].

We focused on the multiple gradients of Wg pathway activation in the adult intestine that demarcate all major boundaries between compartments. Importantly, Wg target gene activation in these gradients requires the direct and continuous activation of Wg signaling both during development and in adulthood [28,37]. Herein, we found that near Wg-producing cells at two distinct intestinal boundaries, tethered Wg is capable of activing target gene expression at levels equivalent to or greater than that induced by wild-type Wg. These findings support the previous conclusion that the NRT-Wg can activate signaling at the source of synthesis [13,19]. However, the spatial range of Wg pathway activation is reduced in *NRT-Wg* intestines, indicating that Wg tethering prevents the activation of target gene expression at a distance from the source. Consequently, Wg tethering in the developing adult gut manifests in many defects: both epithelial and muscle layers at compartment boundaries are improperly patterned, cell fate and proliferation within intestinal compartments are deregulated, the normal folds in the gut tube are lost and ectopic twists develop, signaling crosstalk with the Dpp pathway is diminished, and viability and fitness are reduced. These defects are phenocopied by Wg pathway inhibition in the intestinal epithelium, indicating that Wg secretion and movement are critical for proper Wg pathway activation during adult gut development. Therefore, this work identifies a physiological context in which the requirement for the direct and long-range action of Wg in tissue patterning is unambiguous, supporting conclusions from previous studies in the larval wing disc [11,13].

How does Wg tethering result in dichotomous effects on tissue patterning of the adult intestine versus the larval wing disc? At least one property of the wing disc complicates the conclusion that the long-range action of Wg is dispensable for patterning. *wg* is expressed in complex and transient patterns during development of the wing disc, initially in all cells throughout the wing pouch and subsequently restricted in later stages to the dorsoventral boundary. This feature may have inadvertently resulted in the widespread perdurance of tethered Wg throughout the late larval wing pouch [20], as the NRT-Wg protein may be more stable than wild-type Wg [72]. Thus, it remains possible that a requirement for the long-range action of Wg in the wing disc was masked in previous experiments by the unintended ubiquitous expression of tethered Wg. In contrast, the spatially restricted expression of *wg* to compartment boundaries in the intestinal epithelium is observed not only in the developing adult intestine, but also at earlier larval stages [28], mitigating the concern that NRT-Wg persisted inadvertently in Wg-receiving cells during development of the adult intestine.

If one accepts the conclusion that the long-range action of Wg is essential in the adult intestine but dispensable in the wing disc, then it remains possible that these context-specific *NRT-Wg* phenotypes result from the inherently unique properties of these different tissues. For example, the Wg-receiving cell population, as measured by Wg target gene activation, extends for at least 60 cell diameters from the MHB in the adult posterior midgut [37], but only 15 to 20 cell diameters from the dorsoventral boundary in the larval wing disc [13,20]. Despite its tethering, NRT-Wg might retain an ability to act over short distances, utilizing previously proposed modes of extracellular transport that include cytonemes [73,74,75,76,77], exosomes [78,79,80,81], or via transcytosis [4,82,83]. Moreover, similar to the mammalian intestinal epithelium [84], cell division and subsequent migration might contribute to NRT-Wg distribution. Thus, the range of NRT-Wg movement may suffice for patterning the wing disc but not the intestine. Given the presence of a larger Wg-receiving cell population, the adult intestine may serve as a better model for the role of the long-range action of Wnt in the patterning of larger tissues that include vertebrate limbs and the anteroposterior embryonic axis during mouse gastrulation [85].

Alternatively, the different *NRT-Wg* effects in the wing disc versus adult intestine could result from ‘cellular memory’, a model that proposed persistent activation of Wg target genes in prospective wing cells and their descendants distant from the dorsoventral boundary in the *NRT-Wg* wing disc [19]. In this memory model, the activation of target genes is induced not only upon exposure to a ligand (such as Wg or Dpp), but also after the ligand is no longer present [8]. However, previous studies demonstrated that Wg target gene expression is restricted to the immediate neighbors of cells expressing *NRT-Wg* and that target gene activation in cells distant from the Wg source is dependent on their continuous ability to receive Wg. These findings do not support a memory model, which remains a subject of ongoing debate [11,13,20,86]. Importantly, in contrast with the larval wing disc, adult gut development begins with histolysis of the larval gut [87]; both the epithelium and musculature of the adult intestine are largely reformed during pupation [45,88]. This unique developmental process mitigates the issue of cellular memory. Indeed, our findings indicate that the tethering of Wg during formation of the adult intestine, and not prior, is sufficient to recapitulate the *NRT-Wg* intestinal phenotypes.

Together, our findings document the consequences for adult intestinal development that result from Wg tethering, support previous studies that revealed the requirement for the long-range action of Wg [11,13], and call for assessment of the different physiological contexts in which this long-range action directs tissue patterning and homeostasis. Identification of additional tissues in which the inhibition of Wg movement disrupts signaling, including those reported recently [48,89,90], may uncover the general principles that determine the contexts in which the long-range action of Wnt is essential. Studies in the Drosophila intestine may also be relevant for the mammalian intestine, which is similarly divided into distinct compartments and sub compartments, and dependent on Wnt expression for this regionalization [91,92]. Thus, future work may reveal the extent to which the long-range action of Wnt is required for patterning of both invertebrate and vertebrate tissues, and its potential roles in diseases states.

## Methods

**Fly stocks:** *fz3-RFP* [43], *fz3-GFP* [42], *nkd-lacZ* [41], *GBE-Su(H)-lacZ* [25], *Dad-GFP* [56], *tkv-lacZ* (BDSC#111191) [93], *dpp-lacZ* (obtained from Dr. Stacey Ogden) [94], *wg{KO; NRT-Wg-HA}* and *wg{KO; FRT Wg FRT NRT-Wg-HA}*[19], *wg*^*CX4*^ (BDSC#2980) [95], *pygo*^*S123*^ (BDSC#7209) [58], *dsh*^*3*^ (BDSC#6331) [59], *wntless*^*EY01593*^ (BDSC#13563) [68], *Df(3L)Lxd6* (BDSC#89), *Apc2*^*19*.*3*^ *Apc1*^*Q8*^ [96].

**Controls**: *wg{KO*, *Wg-HA}* [19], *Canton S*, *FRT19A* (BDSC#1709) or *FRT40A* (BDSC#1835) stocks were used as wild-type controls unless otherwise specified.

**MARCM lines:** MARCM 19A: *hs-flp tub-Gal80 FRT19A; tub-Gal4 UAS-mCD8*::*GFP/SM6^TM6B* [97].

MARCM 82B: *y w hs-flp UAS-CD8*::*GFP; tub-Gal4 FRT82B tub-Gal80/TM6B* [98].

MARCM 40: *hsflp; FRT40 tub-Gal80; tub-Gal4 UAS-GFP* [99].

**Gal4 drivers:** *wg*^*ts*^ (*wg{KO*, *Gal4}*, *tub-Gal80*^*ts*^) [19]), *Myo*^*ts*^ (*Myo1A-Gal4*, *tub-Gal80*^*ts*^) [100], *cad*^*ts*^ (*cad-Gal4*; *tub-Gal80*^*ts*^) (derived from BDSC#3042), *dve*^*ts*^ (*dve-Gal4*; *tub-Gal80*^*ts*^) (derived from DGRC#113–273).

**UAS lines:** *UAS-wg RNAi#1* (VDRC#104579; construct ID: 108857), *UAS-wg RNAi#2* (BDSC#32994), *UAS-wntless RNAi#1* (VDRC#5214; construct ID: 2418), *UAS-wntless RNAi#2* (VDRC#103812; construct ID: 101700), *UAS-yellow RNAi* (BDSC#64527), *UAS-porcupine RNAi* (VDRC#9150; construct ID: 3424), *UAS-notum* [49], *UAS-GFP-lacZ* (BDSC#6452), *UAS-ΔN1 dTCF* [51], *UAS-wg* (BDSC#5919) [51], *UAS- NRT-wg-HA* [13]

**Fly husbandry:** Neither homozygous *NRT-Wg* animals, nor *NRT-Wg/ wg*^*null*^ transheterozygotes survived to adulthood at 25°C. Both genotypes survived if the temperature was lowered to 21–22°C to allow for increased developmental time.

### Immunohistochemistry

Primary antibodies: chicken anti-GFP (Abcam, ab13970, 1:10000), rabbit anti-GFP (Invitrogen, A11122, 1:500), mouse anti-GFP (Invitrogen, A11120, 1:500), rat anti-HA (Roche, 11867431001, 1:100), mouse anti-β-gal (Promega, Z3781, 1:500), rabbit anti-β-gal (MP Biomedicals, 08559761, 1:5000), rabbit anti-dsRed (Clontech, 632496, 1:500), rabbit anti-phospho-histone H3 (Ser10) (Millipore, 06–570, 1:1000), mouse anti-Arm (Developmental Studies Hybridoma Bank (DSHB) N2 7A1, 1:20) [101], mouse anti-Prospero (DSHB, MR1A, 1:20) [102], mouse anti-Cut (DSHB, 2B10, 1:20) [103], mouse anti-α-actinin (DSHB, 2G3-3D7, 1:20) [104], mouse anti-Fas III (DSHB, 7G10, 1:20) [105], mouse anti-Discs large (DSHB, 4F3, 1:20) [106], Alexa Fluor 647 phalloidin (Life Technologies, A22287, 1:500), Alexa Fluor 488 phalloidin (Abcam, ab176753, 1:2000), Alexa Fluor 555 phalloidin (Life Technologies, A34055, 1:500), DAPI (Sigma, D9542, 1:500), rabbit anti-cleaved Drosophila Dcp-1 (Asp216) (Cell Signaling, 9578, 1:100), rabbit anti-MHC (myosin heavy chain) (obtained from Dr. Dan Kiehart, 1:500) [107], rabbit anti-labial (obtained from Dr. Benjamin Ohlstein, 1:2000) [99]. Secondary antibodies: goat or donkey Alexa Fluor 488 or 555 conjugates at 1:400 (Life Technologies), and goat or donkey Cy5 conjugates at 1:200 (Life Technologies/Jackson Immunochemicals).

Intestines from adult females at 2 to 3 days post-eclosion were dissected in PBS and fixed in 4% paraformaldehyde in PBS for 45 minutes at room temperature, then washed with PBS, 0.1% Triton X-100, followed by incubation in PBS, 0.1% Tween-20 and 10% BSA for 1 hour at room temperature. The samples were then incubated with primary antibodies at 4°C overnight in PBS, 0.5% Triton X-100. Samples were stained with secondary antibodies for 2 hours at room temperature. Larval intestines were fixed in 4% paraformaldehyde in PBS for 20 minutes at room temperature and immunostained in the process similar for the adult intestine, with the exception that incubation with primary antibodies was in PBS, 0.1% Triton X-100. Specimens were stained with DAPI (2μg/ml) and mounted in Prolong Gold (Invitrogen). Fluorescent images were captured on a Nikon A1RSi confocal microscope. Images were processed using Adobe Photoshop / Illustrator software.

### Survival assay

*wg{KO*, *NRT-Wg-HA}* and *wg{KO*, *Wg-HA}* flies were collected 2 to 3 days post-eclosion. Two different dietary regimens were chosen: flies were either reared at 25°C on standard cornmeal, molasses and yeast medium for 14 days or reared at 29°C for 5 days with filter paper soaked with a 1% sucrose solution. Each day, the flies were transferred to new vials, and the number of survivors was recorded. A survival graph was plotted for each group. Similarly, *notum*-overexpressing flies were collected alongside controls at 1 to 2 days post-eclosion and survival till the following day was documented.

### Clonal analysis

Mitotic clones were generated using the MARCM system [108]. Clones were induced in second instar larvae by a single 2 to 3 hour heat shock at 37°C and examined 1 to 2 days after eclosion. Clones in the posterior and middle midguts were analyzed.

### Transgene expression using temperature-sensitive Gal4

The crosses between lines expressing Gal4 and lines carrying UAS-RNAi transgenes, *UAS*-*notum* or *UAS*-*dNTCF* were maintained at 18°C and transferred to new vials every other day. To activate RNAi expression during development, either 3^rd^ instar larvae or flies at the larvae/pupae transition were shifted from 18°C to 29°C (Day 6 or 8 for *notum*, *dNTCF* and *wntless* RNAi and Day 8 or 10 for *wingless* RNAi). Progeny were collected within 2 days of eclosion and maintained at 29°C for one more day before examination. For overexpression of *UAS-NRT-Wg* and gene conversion, animals were shifted from 18°C to 29° C as 3^rd^ instar larvae.

### Quantification and statistics

The MMG and PMG were identified based on *fz3-RFP* and phalloidin staining patterns. To measure the length of PMG and MMG, lines were drawn along the longitudinal muscle fibers (based on phalloidin staining), and compartment length was measured using NIS-Elements software. Quantification of *fz3-GFP* intensity was performed with image analysis tools in NIS-Elements software. Z-stack images were obtained for regions near the MHB and the anterior boundary of MMG (AB-MMG), and maximum intensity projections (max-IP) were generated. For the MHB, the max-IP images were magnified threefold, boxes of defined size were drawn inside *fz3-GFP-*expressing cells adjacent to the MHB and mean intensity within the box was measured with image analysis tools in NIS-Elements software. These measurements were obtained for five randomly selected cells adjacent to the MHB for each sample and were repeated in no less than 10 samples. Similar measurements were performed for the AB-MMG. Quantification of NRT-Wg-HA was performed similarly, with no less than 10 samples. For quantification of proliferation, the total number of pH3-positive cells in the posterior midgut of the indicated genotypes was counted. All statistical tests were performed using Prism (GraphPad Software, USA).

## Supporting information

S1 Fig The spatial range of Wg target gene activation is reduced by Wg tethering. **(A-A’)** Wg protein is present at the MHB. **(B-C)** The spatial range of *fz3-RFP* is reduced in *NRT-Wg* intestines. *fz3-RFP* is also detected in progenitor cells; however, with the exception of the posterior terminal midgut, this expression is not dependent on Wg signaling under homeostatic conditions [28]. **(D-E)** Supernumerary progenitor cells, as revealed by expression *10xStat-GFP*, in *NRT-Wg* posterior midguts. **(F-G’)** The MHB and the majority of the pylorus are lost when Wg is tethered. wild-type Wg: *wg{KO*, *Wg-HA}*; tethered Wg: *wg{KO*, *NRT-Wg-HA}*. Anterior, left. Orange arrow marks the MHB. Green arrow marks the posterior boundary of the pylorus. Scale bars: (A-A’) 25 μm, (B-C) 50 μm, (D-E) 25 μm, (F-G’) 100 μm.
(TIF)

S2 Fig Epithelial defects are not present in the *NRT-Wg* larval intestine. **(A-B’)** The epithelial transition zone of the MHB in the *NRT-Wg* larval intestine as revealed by Fas3 and DAPI. **(C-D”)** AMP clusters anterior to the MHB in *NRT-Wg* larval guts as revealed by Arm staining. wild-type Wg: *wg{KO*, *Wg-HA}*; tethered Wg: *wg{KO*, *NRT-Wg-HA}*. Anterior, left. Arrow marks the MHB. Scale bars: (A-B’) 25 μm, (C-D”) 50 μm.
(TIF)

S3 Fig The short signaling range observed in NRT-Wg guts is not due to reduced signaling activity at the source. **(A-B’)** Wg protein at MHB in *Wg-HA* and *NRT-Wg* intestines. Homozygous *Wg-HA* guts were analyzed, but since *NRT-Wg* homozygotes have defects at the MHB, *NRT-Wg* heterozygotes were analyzed. **(C)** Quantification of *HA* levels adjacent to the MHB **** P<0.001 (t-test). **(D-E’)** Defects in the MHB epithelial transition zone are not rescued by overexpression of *UAS*-*NRT-Wg-HA*. **(F)** Quantification of *fz3-GFP* levels adjacent to the MHB or adjacent to the anterior border of the MMG (AB-MMG). **** P<0.001, n.s.: not significant (t-test). Flipped in *NRT-Wg-HA*: *wg*^*ts*^ (*wg{KO*, *Gal4}*, *tub-Gal80*^*ts*^)/ *wg{KO*,*FRT Wg FRT NRT-Wg-HA}; UAS-FLP*. Overexpressed *NRT-Wg-HA*: *wg*^*ts*^ (*wg{KO*, *Gal4}*, *tub-Gal80*^*ts*^)/ *wg{KO; FRT Wg FRT NRT-Wg-HA*, *UAS- NRT-Wg-HA};UAS-FLP/TM2*. Anterior, left. Arrow marks the MHB. Scale bars: (A-B) 25μm, (D-E’) 25 μm.
(TIF)

S4 Fig Inhibition of Wg, but not its overexpression, phenocopies Wg tethering at the MHB. **(A)** The *cad-Gal4* driver drives strong expression at the MHB and in the posterior half of the PMG. **(B)** Overexpression of *notum* or *dominant-negative TCF* results in ISC over proliferation in the PMG, revealed by pHH3. **** P<0.001 (t-test). **(C-F”)** Epithelial and muscle patterning of the MHB is preserved upon *wg* overexpression. Anterior, left. Orange arrow marks the MHB. Silver arrows mark the anterior and posterior boundaries of MMG. Scale bars: (A) 100 μm, (C-D) 500 μm, (E-F”) 25 μm.
(TIF)

S5 Fig Wg signaling is required for *dpp* expression in the distal posterior midgut. **(A-C’)** Loss of Wg signaling in *pygo* null mutant clones results in loss of *dpp* expression in the posterior midgut. In *pygo* clones at a distance from the MHB (yellow square, **A**, higher magnification in **B** and **B’**) *dpp-lacZ* expression is lost, whereas clones near the MHB (blue square, **A**, higher magnification in **C** and **C’**) retain *dpp-lacZ* expression. **(D-E”)** Hyperactivation of Wg signaling results in ectopic *dpp* expression outside the normal Dpp gradient. Wg signaling is hyperactivated in *Apc1 Apc2* double null mutant clones. Clones that fall in the low gradient region (yellow square, **D**, higher magnification in **D’** and **D”**) induce high expression of *dpp-lacZ*. *dpp-lacZ* expression is not increased in clones that reside within the high *dpp-lacZ* gradient region (blue square, **E**, zoom-in in **E’** and **E”**). Arrow marks the MHB. Scale bars: (A, D, E) 25 μm.
(TIF)

S6 Fig Wg signaling promotes *tkv* expression and Dpp target gene activation near the MHB. **(A-A”)** *tkv-lacZ*, a transcription reporter for a Dpp pathway receptor, exhibits graded expression at the MHB, which is nearly lost in *dsh* mutant clones near the MHB (yellow square, **A**, higher magnification in **A’** and **A”**). **(B-B”)** Expression of *Dad-GFP*, a transcriptional reporter for the Dpp pathway target gene *Dad*, is lost in *dsh* mutant clones (yellow square, **B**, higher magnification in **B’** and **B”**). Scale bars: (A and B) 25 μm.
(TIF)

S7 Fig Wg tethering disrupts copper cell fate specification. **(A-B)** Labial is expressed specifically in copper cells. In *NRT-Wg* midguts, only a few Labial-marked cells are detected, and are restricted to the anterior MMG boundary. **(C-F)** *NRT-Wg/wg*^*null*^ phenocopies *NRT-Wg* homozygotes: decreased MMG size and decreased number of Cut-positive copper cells. wild-type Wg: *wg{KO*, *Wg-HA}*; tethered Wg: *wg{KO*, *NRT-Wg-HA}*. Anterior, left. Orange arrow marks the anterior boundary of the MMG. Silver arrow marks the posterior boundary of the MMG. Scale bars: (A, B, E, F) 25 μm, (C-D) 100 μm.
(TIF)

S8 Fig Attenuation of Wg signaling phenocopies Wg tethering at the MMG. **(A)** *cad-Gal4* drives expression at the anterior and posterior boundaries of the MMG (though weaker than at the MHB). **(B)** *dve-Gal4* drives strong expression in the entire MMG. **(C-F)** Overexpression of *notum* with *cad-Gal4* results in malformation of the MMG, and disrupts patterning of muscles overlying the MMG. **(G-H)** Overexpression of *notum* with *dve-Gal4* results in difficulty discerning the MMG, with only one remaining *fz3-RFP* enriched boundary, and only a few remaining copper cells. * An ectopic twist is formed anterior to this region. **(I-J)** Overexpression of *wg* with *cad-Gal4* also results in malformation of the MMG. Anterior, left. Orange arrow marks the anterior boundary of the MMG. Silver arrow marks the posterior boundary of the MMG. Scale bars: (A-B) 100 μm, (C, D, G-J) 50 μm, (E-F) 25 μm.
(TIF)

S9 Fig Inhibition of Wg signaling at the MMG results in decreased MMG size. **(A-E)** RNAi-mediated knockdown of *wg* or *wls* reduces MMG size. To rule out off-target effects, two independent RNAi lines were tested for each gene. Quantification in **D and E**, **** p<0.001 (t-test). **(F-G)** *wls* mutants display reduced MMG size. Quantification in **H**, **** p<0.001, (t-test). Anterior, left. Orange arrow marks the anterior boundary of the MMG. Silver arrow marks the posterior boundary of the MMG. Scale bars: (A, B, D, F, G) 500 μm.
(TIF)

S10 Fig Either tethering Wg or diminishing Wg activity in the intestinal epithelium reduces fitness. With standard food **(A)** or a sucrose only diet **(B)**, *NRT-Wg* mutant lifespan is reduced by comparison to controls. **(C-D)** An abnormally large crop in *NRT-Wg* intestines. **(E)** Wg pathway inhibition in the intestinal epithelium reduces fitness. Anterior, left. wild-type Wg: *wg{KO*, *Wg-HA}*; tethered Wg: *wg{KO*, *NRT-Wg-HA}*. Number counted for survival assay: **(A and B)** wild-type Wg, n = 100; tethered Wg, n = 100; **(E)** *UAS-GFP-lacZ*: n = 34; *UAS-notum*, n = 31; *UAS-dNTCF*, n = 22. Scale bars: (C-D) 200 μm.
(TIF)
