# Supplementary figures and images for: Essential long-range action of Wingless/Wnt in adult intestinal compartmentalization
Source: PLoS Genet. 2019 Jun 13;15(6):e1008111. doi: 10.1371/journal.pgen.1008111 (PMC6563961; doi:10.1371/journal.pgen.1008111)

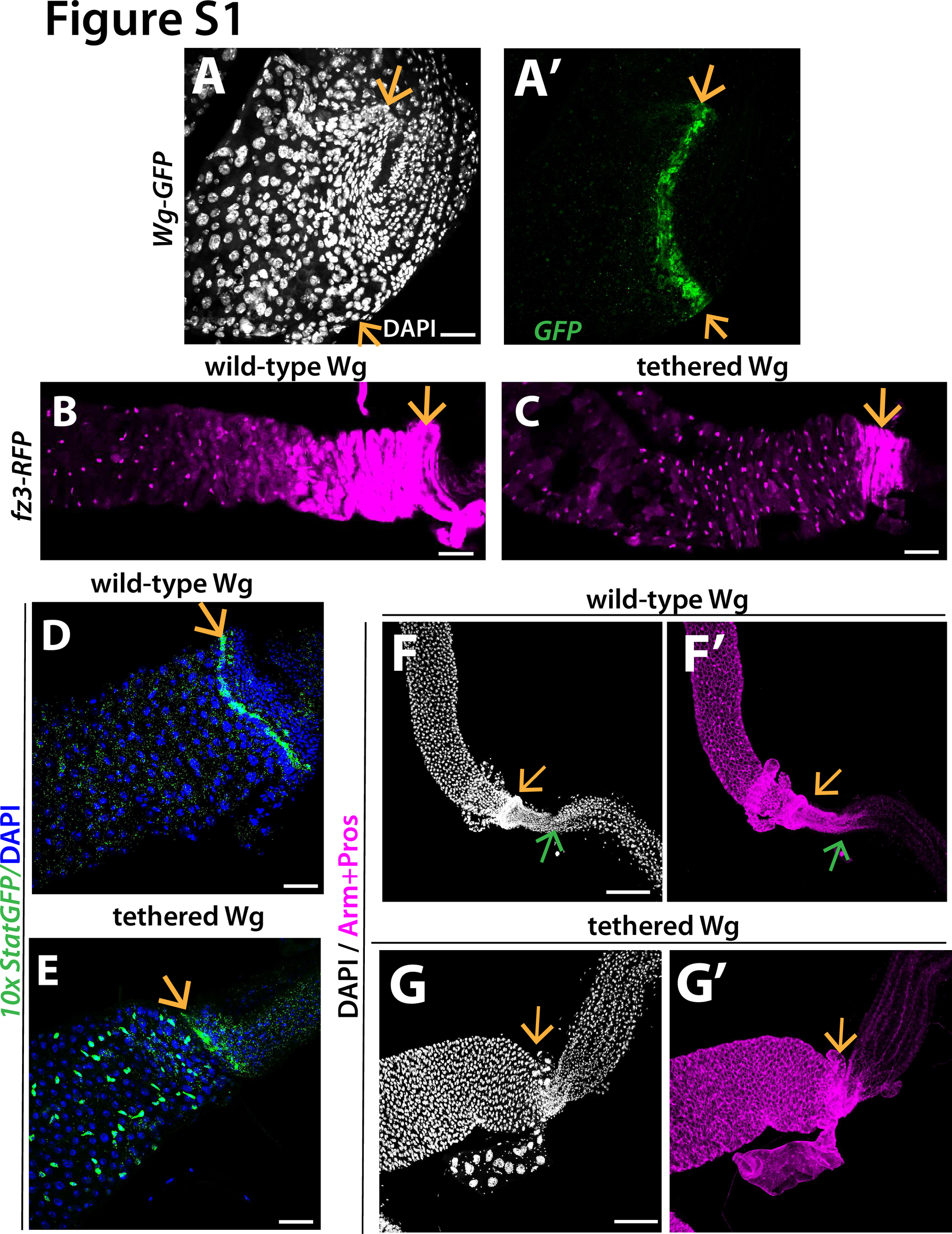

Supplement: S1 Fig — (A-A’) Wg protein is present at the MHB. (B-C) The spatial range of fz3-RFP is reduced in NRT-Wg intestines. fz3-RFP is also detected in progenitor cells; however, with the exception of the posterior terminal midgut, this expression is not dependent on Wg signaling under homeostatic conditions [28]. (D-E) Supernumerary progenitor cells, as revealed by expression 10xStat-GFP, in NRT-Wg posterior midguts. (F-G’) The MHB and the majority of the pylorus are lost when Wg is tethered. wild-type Wg: wg{KO, Wg-HA}; tethered Wg: wg{KO, NRT-Wg-HA}. Anterior, left. Orange arrow marks the MHB. Green arrow marks the posterior boundary of the pylorus. Scale bars: (A-A’) 25 μm, (B-C) 50 μm, (D-E) 25 μm, (F-G’) 100 μm. (TIF) [file pgen.1008111.s001.tif]

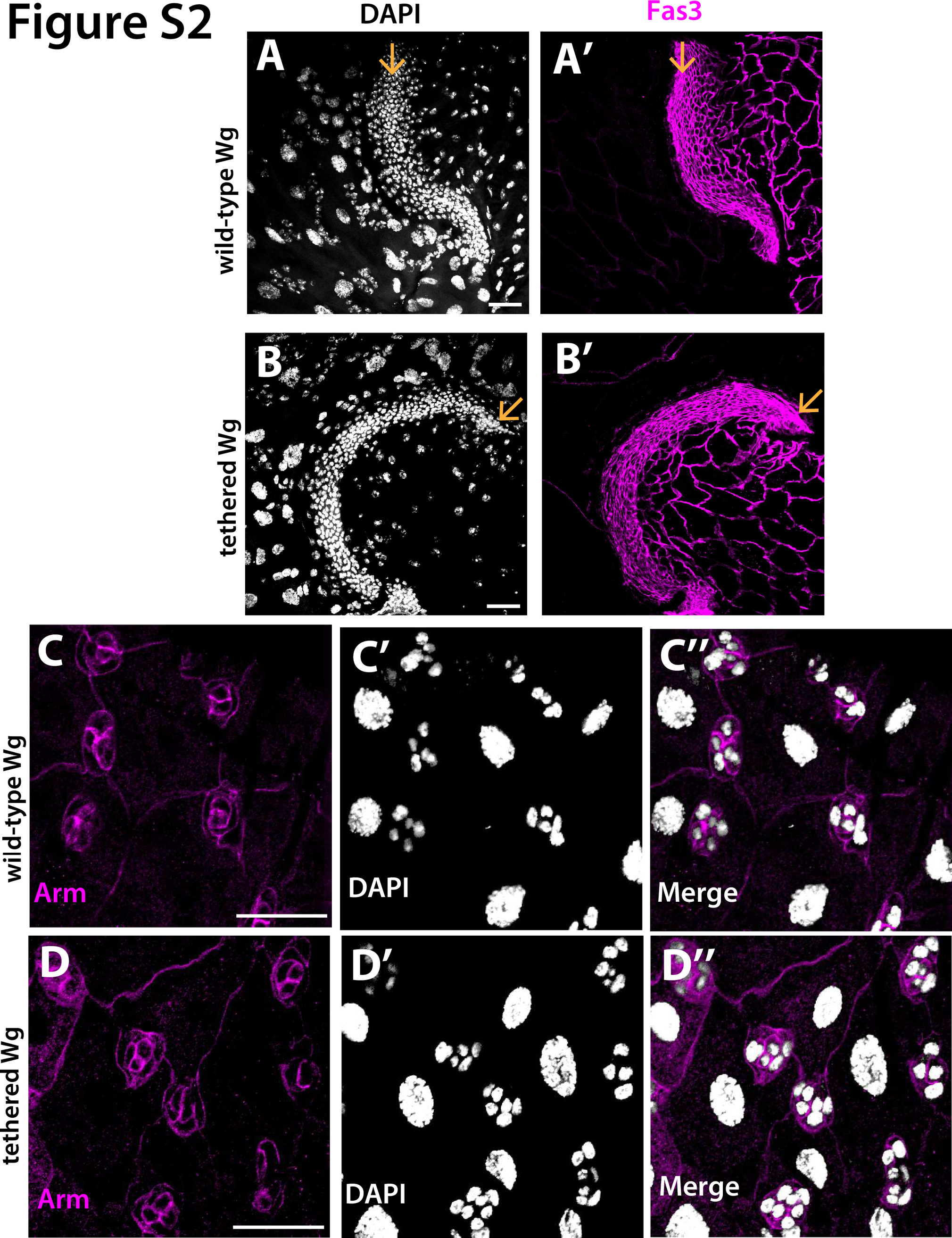

Supplement: S2 Fig — (A-B’) The epithelial transition zone of the MHB in the NRT-Wg larval intestine as revealed by Fas3 and DAPI. (C-D”) AMP clusters anterior to the MHB in NRT-Wg larval guts as revealed by Arm staining. wild-type Wg: wg{KO, Wg-HA}; tethered Wg: wg{KO, NRT-Wg-HA}. Anterior, left. Arrow marks the MHB. Scale bars: (A-B’) 25 μm, (C-D”) 50 μm. (TIF) [file pgen.1008111.s002.tif]

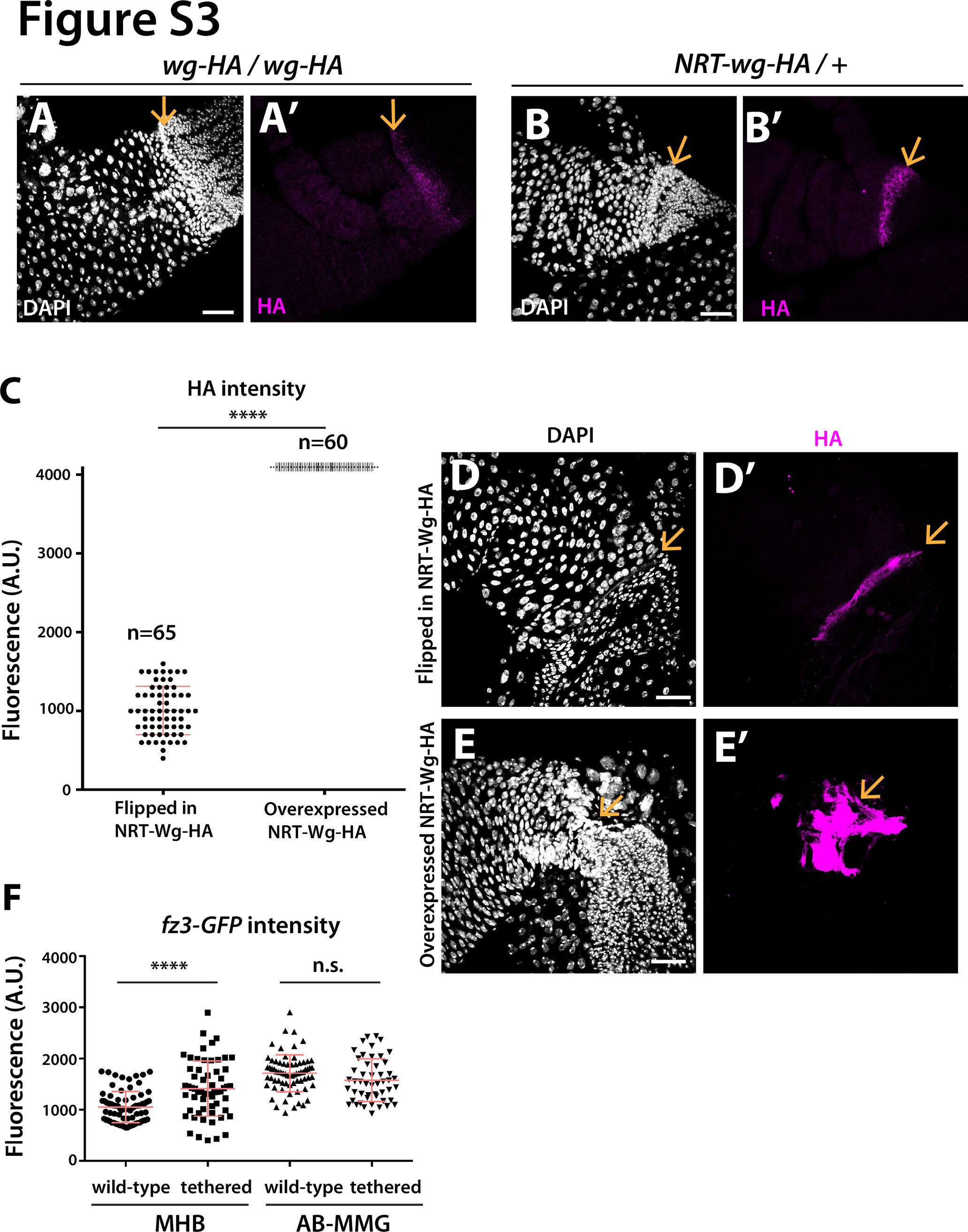

Supplement: S3 Fig — (A-B’) Wg protein at MHB in Wg-HA and NRT-Wg intestines. Homozygous Wg-HA guts were analyzed, but since NRT-Wg homozygotes have defects at the MHB, NRT-Wg heterozygotes were analyzed. (C) Quantification of HA levels adjacent to the MHB **** P<0.001 (t-test). (D-E’) Defects in the MHB epithelial transition zone are not rescued by overexpression of UAS-NRT-Wg-HA. (F) Quantification of fz3-GFP levels adjacent to the MHB or adjacent to the anterior border of the MMG (AB-MMG). **** P<0.001, n.s.: not significant (t-test). Flipped in NRT-Wg-HA: wgts (wg{KO, Gal4}, tub-Gal80ts)/ wg{KO,FRT Wg FRT NRT-Wg-HA}; UAS-FLP. Overexpressed NRT-Wg-HA: wgts (wg{KO, Gal4}, tub-Gal80ts)/ wg{KO; FRT Wg FRT NRT-Wg-HA, UAS- NRT-Wg-HA};UAS-FLP/TM2. Anterior, left. Arrow marks the MHB. Scale bars: (A-B) 25μm, (D-E’) 25 μm. (TIF) [file pgen.1008111.s003.tif]

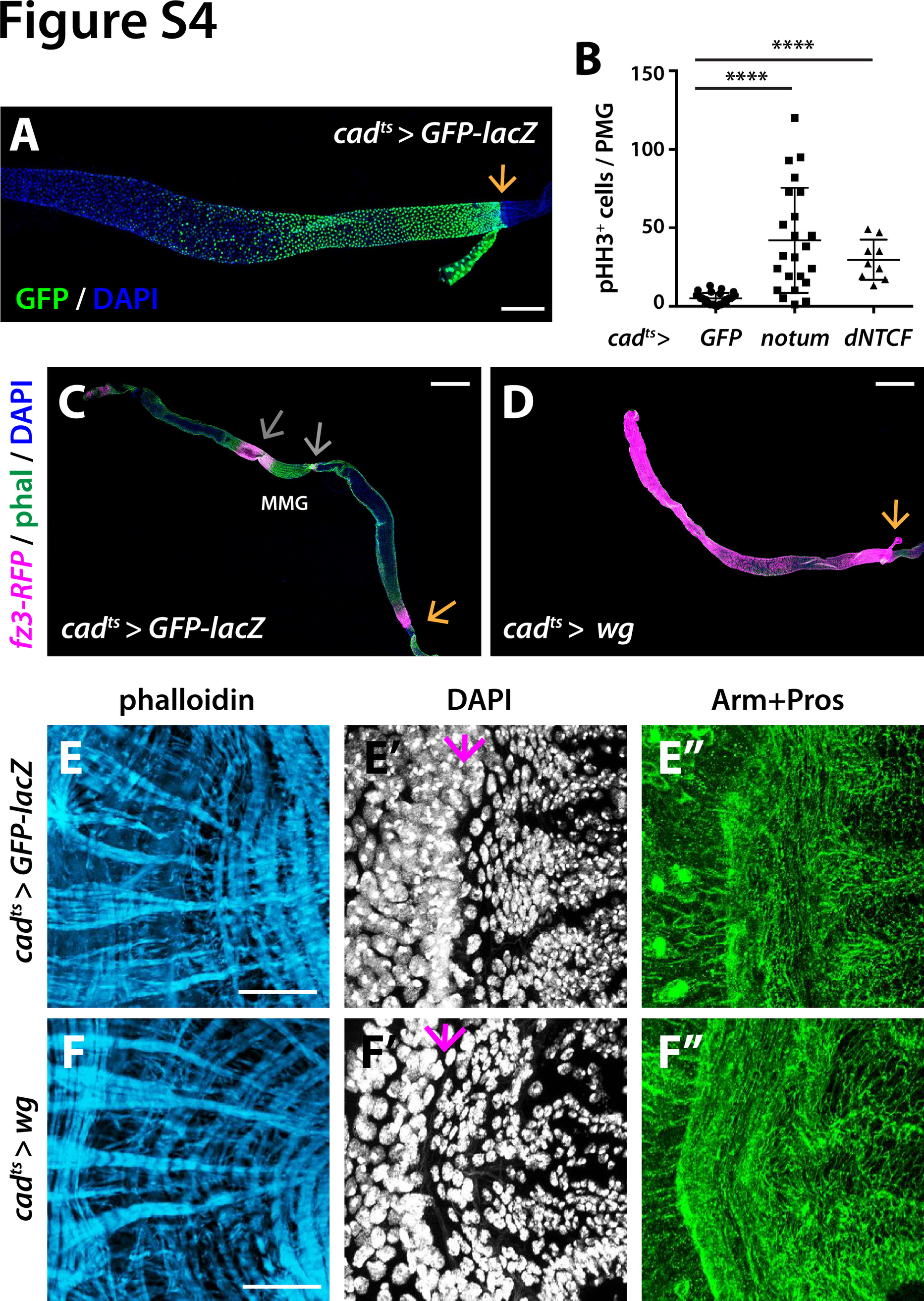

Supplement: S4 Fig — (A) The cad-Gal4 driver drives strong expression at the MHB and in the posterior half of the PMG. (B) Overexpression of notum or dominant-negative TCF results in ISC over proliferation in the PMG, revealed by pHH3. **** P<0.001 (t-test). (C-F”) Epithelial and muscle patterning of the MHB is preserved upon wg overexpression. Anterior, left. Orange arrow marks the MHB. Silver arrows mark the anterior and posterior boundaries of MMG. Scale bars: (A) 100 μm, (C-D) 500 μm, (E-F”) 25 μm. (TIF) [file pgen.1008111.s004.tif]

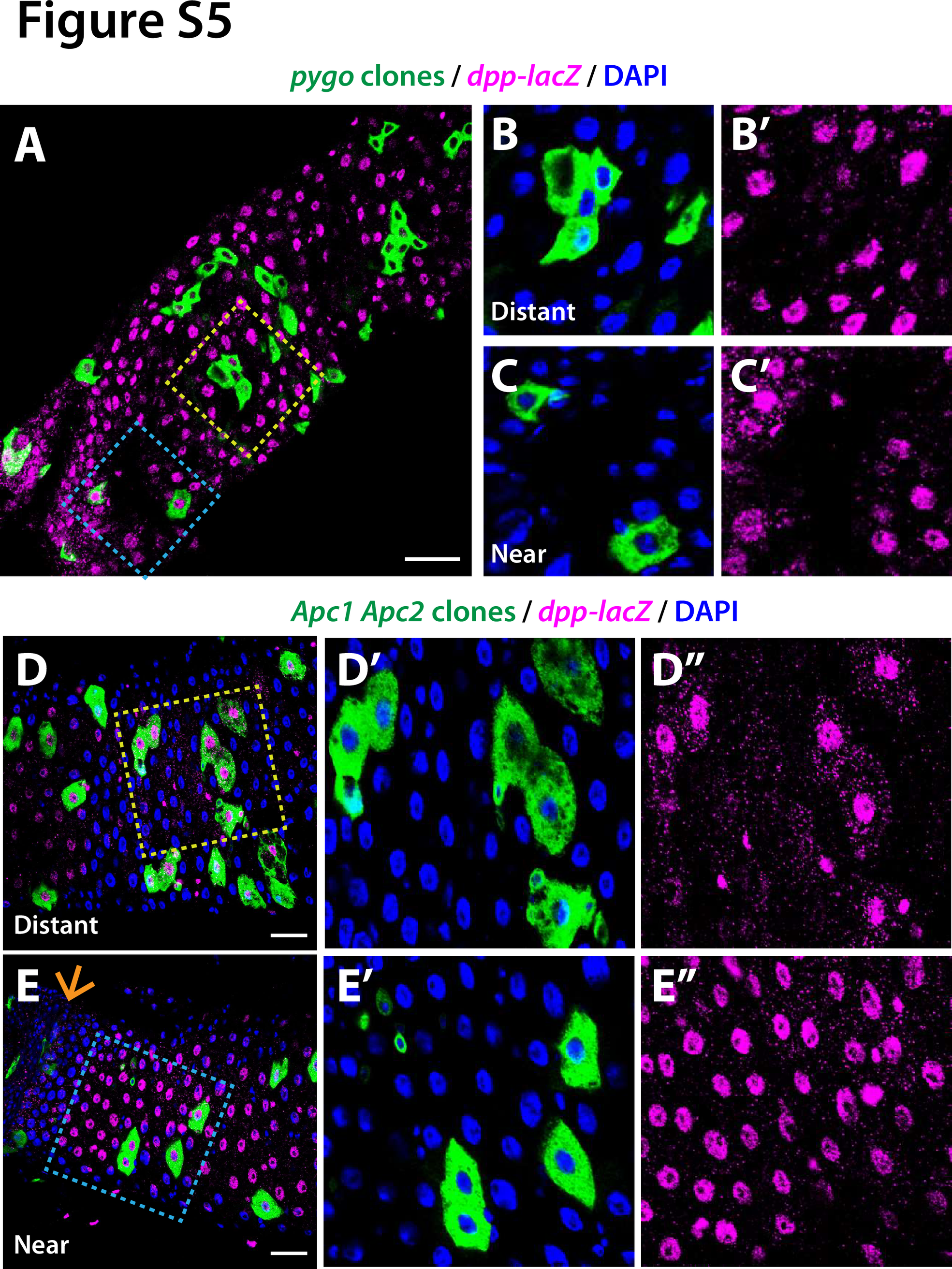

Supplement: S5 Fig — (A-C’) Loss of Wg signaling in pygo null mutant clones results in loss of dpp expression in the posterior midgut. In pygo clones at a distance from the MHB (yellow square, A, higher magnification in B and B’) dpp-lacZ expression is lost, whereas clones near the MHB (blue square, A, higher magnification in C and C’) retain dpp-lacZ expression. (D-E”) Hyperactivation of Wg signaling results in ectopic dpp expression outside the normal Dpp gradient. Wg signaling is hyperactivated in Apc1 Apc2 double null mutant clones. Clones that fall in the low gradient region (yellow square, D, higher magnification in D’ and D”) induce high expression of dpp-lacZ. dpp-lacZ expression is not increased in clones that reside within the high dpp-lacZ gradient region (blue square, E, zoom-in in E’ and E”). Arrow marks the MHB. Scale bars: (A, D, E) 25 μm. (TIF) [file pgen.1008111.s005.tif]

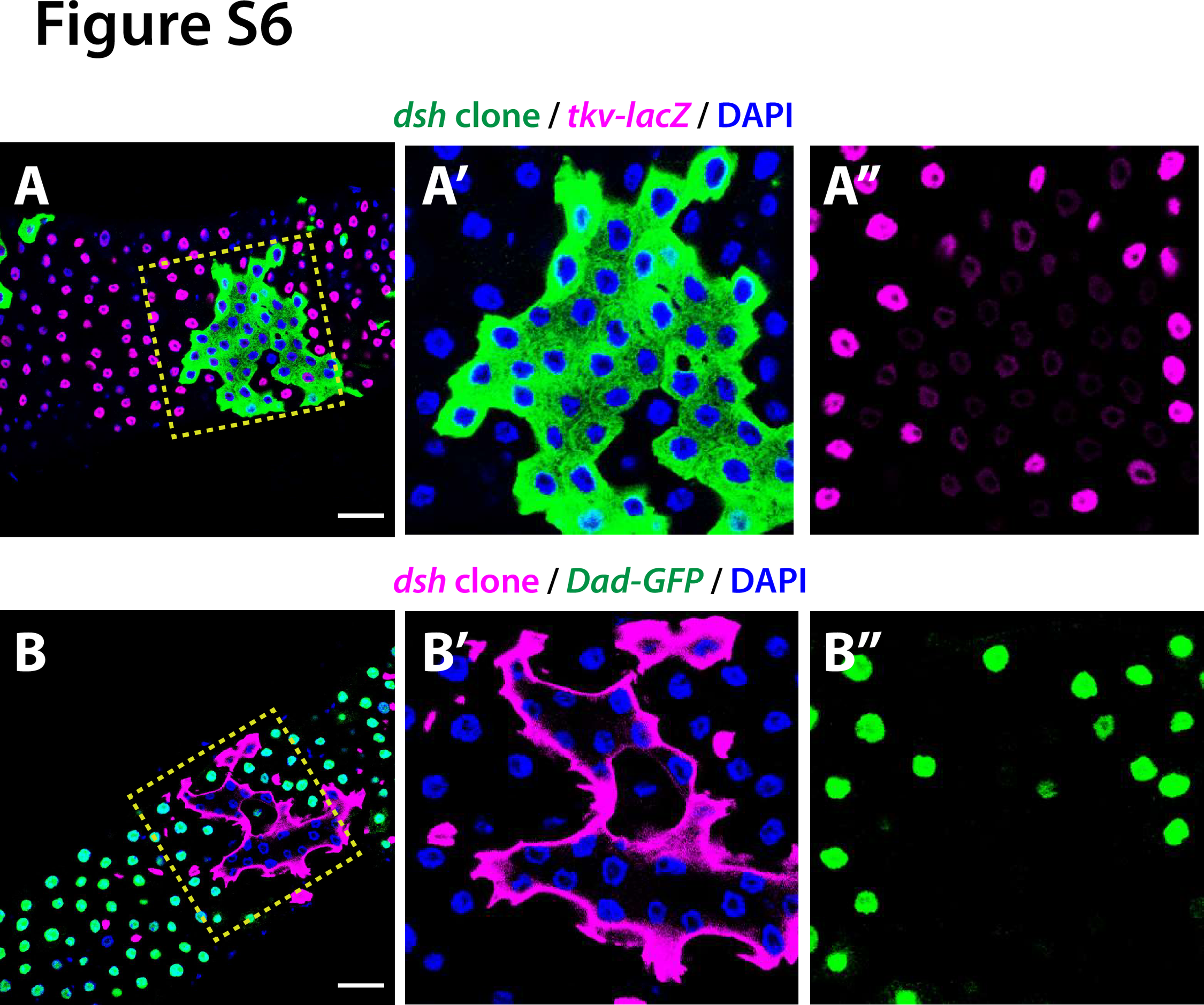

Supplement: S6 Fig — (A-A”) tkv-lacZ, a transcription reporter for a Dpp pathway receptor, exhibits graded expression at the MHB, which is nearly lost in dsh mutant clones near the MHB (yellow square, A, higher magnification in A’ and A”). (B-B”) Expression of Dad-GFP, a transcriptional reporter for the Dpp pathway target gene Dad, is lost in dsh mutant clones (yellow square, B, higher magnification in B’ and B”). Scale bars: (A and B) 25 μm. (TIF) [file pgen.1008111.s006.tif]

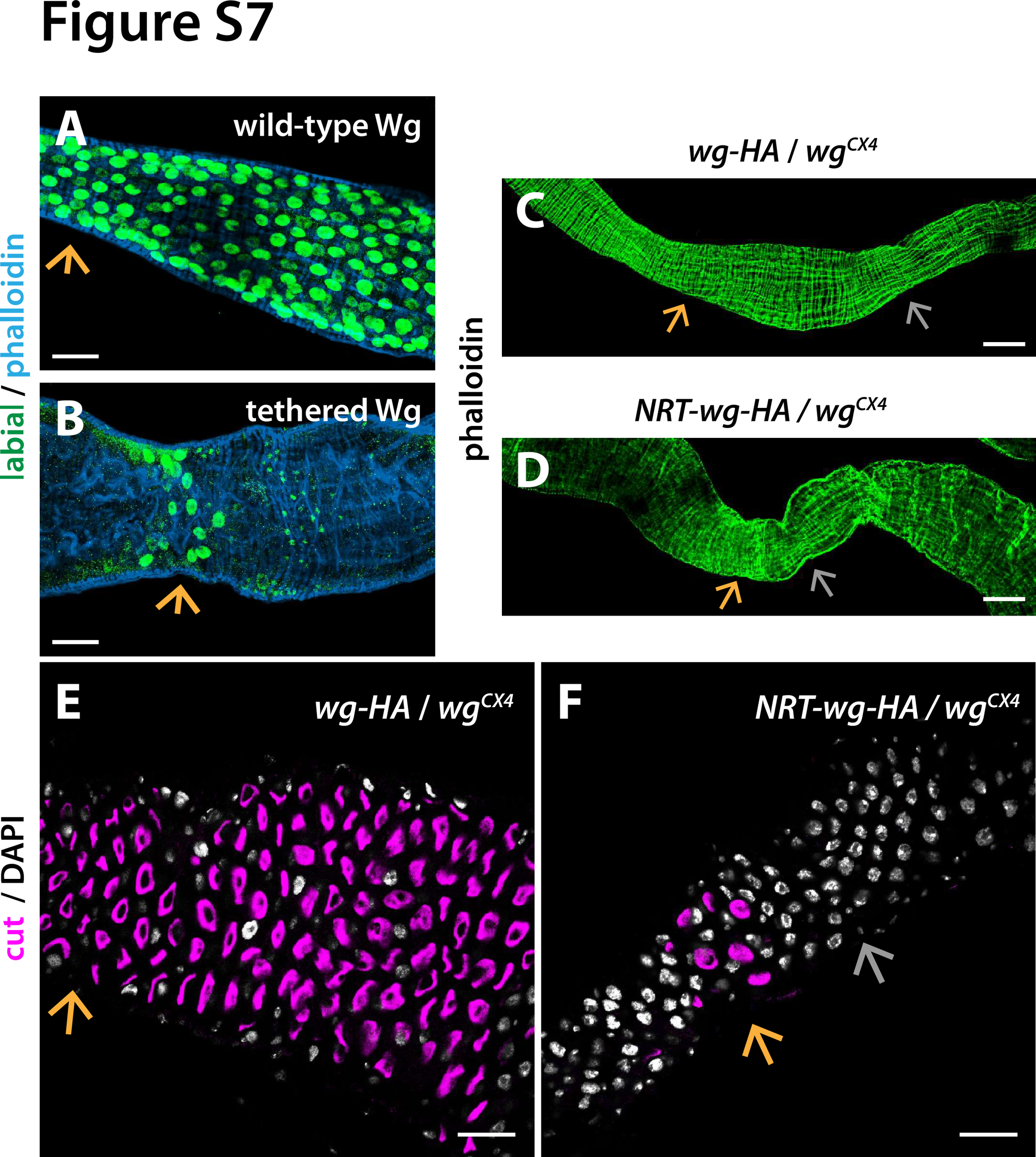

Supplement: S7 Fig — (A-B) Labial is expressed specifically in copper cells. In NRT-Wg midguts, only a few Labial-marked cells are detected, and are restricted to the anterior MMG boundary. (C-F) NRT-Wg/wgnull phenocopies NRT-Wg homozygotes: decreased MMG size and decreased number of Cut-positive copper cells. wild-type Wg: wg{KO, Wg-HA}; tethered Wg: wg{KO, NRT-Wg-HA}. Anterior, left. Orange arrow marks the anterior boundary of the MMG. Silver arrow marks the posterior boundary of the MMG. Scale bars: (A, B, E, F) 25 μm, (C-D) 100 μm. (TIF) [file pgen.1008111.s007.tif]

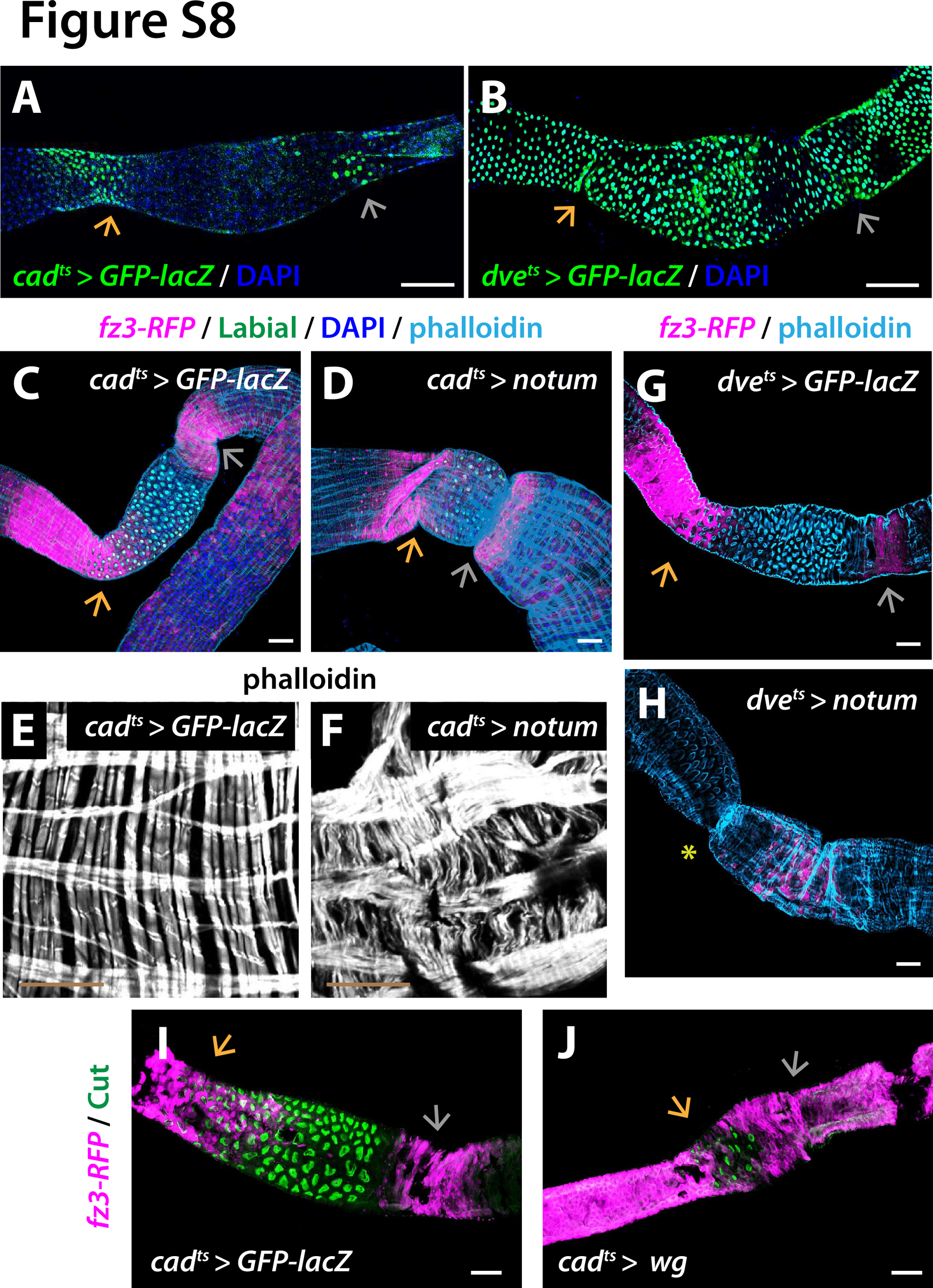

Supplement: S8 Fig — (A) cad-Gal4 drives expression at the anterior and posterior boundaries of the MMG (though weaker than at the MHB). (B) dve-Gal4 drives strong expression in the entire MMG. (C-F) Overexpression of notum with cad-Gal4 results in malformation of the MMG, and disrupts patterning of muscles overlying the MMG. (G-H) Overexpression of notum with dve-Gal4 results in difficulty discerning the MMG, with only one remaining fz3-RFP enriched boundary, and only a few remaining copper cells. * An ectopic twist is formed anterior to this region. (I-J) Overexpression of wg with cad-Gal4 also results in malformation of the MMG. Anterior, left. Orange arrow marks the anterior boundary of the MMG. Silver arrow marks the posterior boundary of the MMG. Scale bars: (A-B) 100 μm, (C, D, G-J) 50 μm, (E-F) 25 μm. (TIF) [file pgen.1008111.s008.tif]

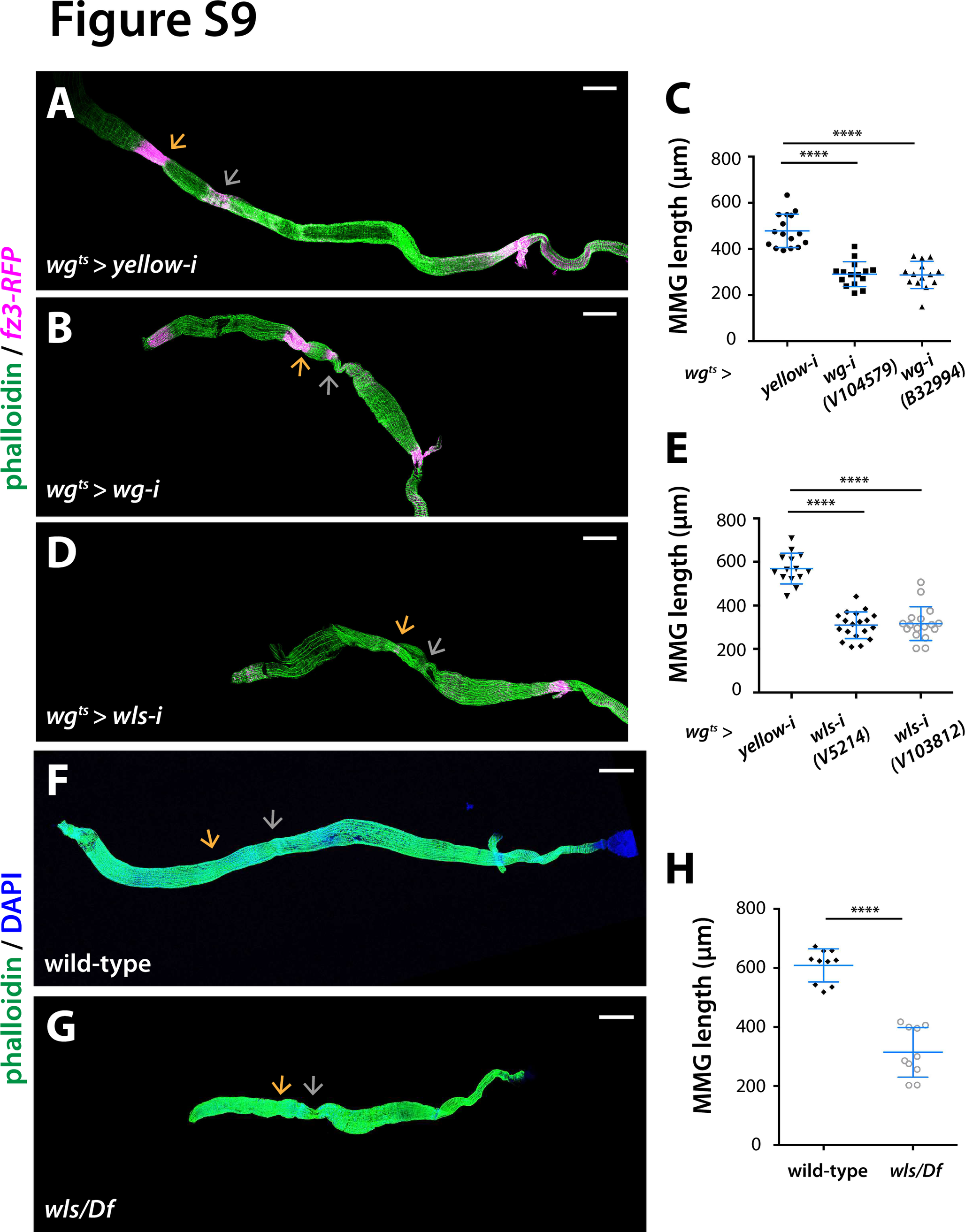

Supplement: S9 Fig — (A-E) RNAi-mediated knockdown of wg or wls reduces MMG size. To rule out off-target effects, two independent RNAi lines were tested for each gene. Quantification in D and E, **** p<0.001 (t-test). (F-G) wls mutants display reduced MMG size. Quantification in H, **** p<0.001, (t-test). Anterior, left. Orange arrow marks the anterior boundary of the MMG. Silver arrow marks the posterior boundary of the MMG. Scale bars: (A, B, D, F, G) 500 μm. (TIF) [file pgen.1008111.s009.tif]

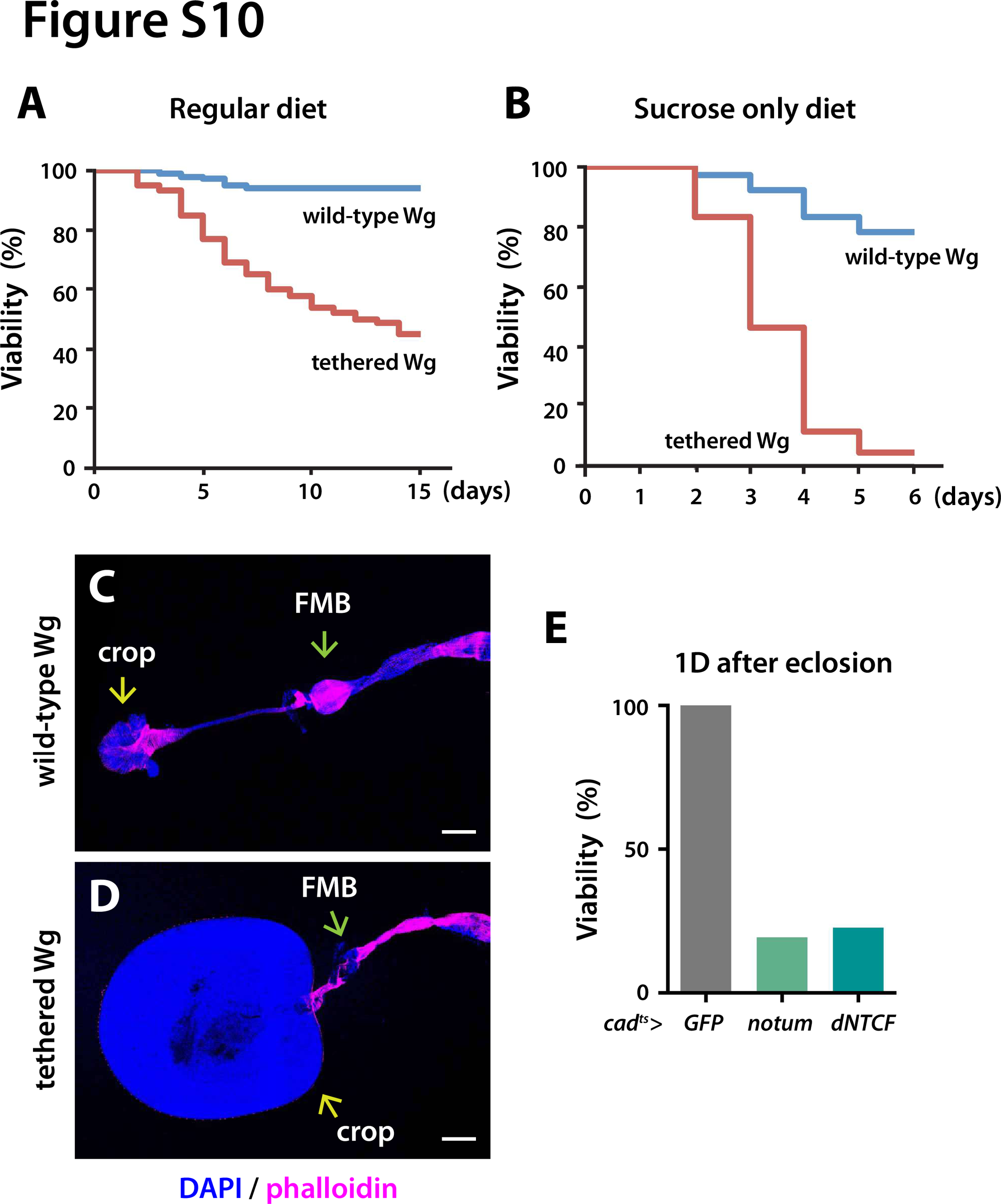

Supplement: S10 Fig — With standard food (A) or a sucrose only diet (B), NRT-Wg mutant lifespan is reduced by comparison to controls. (C-D) An abnormally large crop in NRT-Wg intestines. (E) Wg pathway inhibition in the intestinal epithelium reduces fitness. Anterior, left. wild-type Wg: wg{KO, Wg-HA}; tethered Wg: wg{KO, NRT-Wg-HA}. Number counted for survival assay: (A and B) wild-type Wg, n = 100; tethered Wg, n = 100; (E) UAS-GFP-lacZ: n = 34; UAS-notum, n = 31; UAS-dNTCF, n = 22. Scale bars: (C-D) 200 μm. (TIF) [file pgen.1008111.s010.tif]
